# Breaking Through Barriers: Factors That Influence Behavior Change Toward Leadership for Women in Academic Medicine

**DOI:** 10.3389/fpsyg.2022.854488

**Published:** 2022-05-13

**Authors:** Clara M. Pelfrey, Philip A. Cola, Joshua A. Gerlick, Billie K. Edgar, Sumita B. Khatri

**Affiliations:** ^1^Clinical and Translational Science Collaborative, School of Medicine, Case Western Reserve University, Cleveland, OH, United States; ^2^Department of Design and Innovation, Weatherhead School of Management, Case Western Reserve University, Cleveland, OH, United States; ^3^Cleveland Clinic Lerner College of Medicine at Case Western Reserve University, Cleveland, OH, United States

**Keywords:** women, academic medicine, leadership development, situational awareness, communication, networking, visioning, self-efficacy

## Abstract

Under-representation of women in leadership at Academic Medical Centers (AMCs) is a known challenge such that, in 2021, women made up only 28% of department chairs. AMCs are addressing the dearth of women leaders through targeted programming to create leadership pipelines of qualified women. The FLEX Leadership Development Program at the Case Western Reserve University (CWRU) School of Medicine prepares women faculty for increased leadership opportunities. FLEX includes the opportunity to leverage executive coaching to accomplish individual goals. The FLEX program has the explicit goal of increasing the number of women in visible leadership positions in academic medicine and health sciences. Semi-structured interviews were conducted with 25 graduates from seven FLEX cohorts (2012–2018). Participants reflected diversity in academic rank, terminal degree, racial/ethnic background, years of employment, and institutional affiliation. Interviews consisted of eight questions with additional probes to elicit lived experiences. Analysis consisted of two-stage open- and axial-coding of interview transcripts to understand: What factors facilitated behavior change following FLEX training? The analysis revealed five overarching themes: (1) Communication skills; (2) Self-Efficacy; (3) Networking; (4) Situational Awareness; and (5) Visioning. FLEX graduates reported achieving both personal and professional growth by drawing upon peer networks to proactively seek new leadership opportunities. These results suggest that the enduring benefits of the FLEX Program include improved communication skills, expanded situational awareness and relational capacity, greater self-efficacy and self-confidence, improved networking with an understanding of the value of networking. All these factors led FLEX graduates to have greater visibility and to engage with their colleagues more effectively. Similarly, FLEX graduates could better advocate for themselves and for others as well as paying it forward to mentor and train the next generation of faculty. Finally, participants learned to re-evaluate their goals and their career vision to be able to envision themselves in greater leadership roles. The five factors that strongly influenced behavior change provide valuable constructs for other programs to examine following leadership development training. Ongoing studies include examining successful leadership position attainment, personal goal attainment, and measuring changes in leadership self-efficacy.

## Introduction

Under-representation of women among leadership at academic medical centers (AMCs) is a known challenge. Despite the fact that the percentage of women medical school faculty has increased to 43% in 2021, women remain underrepresented in the national ranks of assistant, associate and full professor (48, 40, and 28%, respectively), and department chair (28%; Association of American Medical Colleges, 2021a). These disparities are attributed to long-standing disadvantages that women in the physician and scientific workforce have endured over time, including: women are not promoted as quickly and do not reach similar levels of leadership positions as men; women receive lower salaries and other compensation despite equal effort, rank and training compared to men; and women receive fewer honors and recognition and are invited to speak less often than men (Freund et al., 2016; Lautenberger and Dandar, 2020). Studies suggest these negative factors contribute to higher rates of attrition among female junior faculty members. Women physician-scientists, who balance clinical practice with teaching and scholarship expectations, have an increased risk of departure compared with colleagues in predominantly clinical or academic domains (Speck et al., 2012). Women also face deep social stereotypes and institutional policies incongruent with parenthood, which further exacerbate gender inequity.

Academic medical centers are addressing the dearth of women leaders through targeted programming to create a leadership pipeline of qualified women (Carr et al., 2017). Programs focusing on leadership skill development improve gender equity by reducing the likelihood of attrition (Chang et al., 2016). The FLEX Leadership Development Program at the Case Western Reserve University (CWRU) School of Medicine provides training and skill development as well as advocacy, networking and professional development that prepares women faculty to develop leadership skills, improve emotional and social competencies, and build capacity in verbal and nonverbal communication. FLEX also includes the opportunity to leverage executive coaching to accomplish individual goals. Qualitative data collected from open-ended surveys captured lived experiences of participants both during training and upon program completion. In addition, semi-structured interviews with 25 graduates between 1 and 8 years after training allowed for *post hoc* reflection on the impact and outcomes of personal leadership development in the months or years after FLEX participation. Each FLEX activity addresses one or more of the challenges women face in navigating AMC environments, as well as aligning individual strengths to achieve personal goals of participants. The FLEX program has the explicit goal of increasing the number of women in visible leadership positions in academic medicine and health sciences. As of 2020, over 127 women faculty have participated in eight different FLEX cohorts since 2012. Here, we present the results of qualitative evaluations of 7 years of the FLEX Program by addressing the following question: (1) What factors facilitated behavior change following FLEX training? This study provides evidence that the FLEX program facilitates behavioral adaptation to help women navigate their work environment to achieve personal and professional leadership goals.

## Background of Flex Program

Under-representation of women and minorities among leadership at AMCs is a known challenge. To address this challenge, Pamela B. Davis, Dean of the CWRU School of Medicine, recommended the development of a professional development program for women faculty at the CWRU School of Medicine. In 2012, she empowered the Women Faculty of the School of Medicine (WFSOM), an organization that provides advocacy, networking, and professional development for women faculty, to create the FLEX Professional Development Program. This program had the explicit goal of increasing the number of women in leadership positions in academic medicine and health sciences. Using focus groups and surveys, the WFSOM steering committee identified several challenges in the SOM that needed to be addressed, including effective mentoring, isolation, and early/mid-career attrition of women faculty. To address these challenges, FLEX was designed to build competencies in communication, leadership, and executive presence to help women faculty prepare for leadership opportunities. Each activity includes women medical professionals and scientists with appointments in CWRU-affiliated institutions, including the VA Medical Center, MetroHealth Medical Center (MHMC), University Hospitals, and the Cleveland Clinic.

The FLEX Program consists of a total of seven full-day workshops and interactive sessions delivered over the course of 7 months. The program curriculum includes leadership skills training and workshops centered on executive presence, building capacity and competency in verbal and nonverbal communication, 360° assessment, DiSC behavior assessment evaluation (Everything DiSC, 2013), serving leader tools, personal productivity, time management, technology-inspired solutions to streamline work, negotiation, resilience, and networking. Participants had the option to meet up to three times with an executive career coach. The final session is a workshop followed by a graduation ceremony and networking event, to which all former FLEX graduates are invited.

## Literature Review

We begin our review by assessing the historical background for the context of our study—divergent demographic trends in which women increasingly represent the majority population in United States medical schools but lag male counterparts as faculty and senior leaders at AMCs. We then consider the particularly salient theory of resonant leadership as it relates to the transition of individuals from the “real” to “ideal” vision of self. We conclude by assessing two cross-cutting theories of knowledge acquisition and behavior change—social cognitive theory, with roots in the fields of psychology, education, and communication, and social network theory, drawn from information systems and management.

### Women in Academic Medicine

Demographic indicators have shifted toward a present majority representation of women matriculants into United States medical schools (Association of American Medical Colleges, 2021b). While representative proportions of women medical school faculty have slightly improved over the last decade, there are still 18% fewer women faculty in medical schools compared to men (Lautenberger and Dandar, 2020, p. 16). Most striking is the lack of senior leadership representation among women hospital CEOs (18%), deans and department chairs in the United States (16%), senior authorship in medical literature (10%), and journal editors-in-chief (7%; Mangurian et al., 2018). Many women find the prestige of being distinguished on the forefront of medical knowledge and the allyship of like-minded intellectual colleagues make the challenge of academic medicine reflect a positive work-lifestyle fit (Borges et al., 2012). However, among women who ultimately choose to leave academic medicine, key factors associated with their departure include a lack of perceived peer leader role models, lacking allyship to support the achievement of professional goals, and frustrations (lack of funding, mentorship support, and protected time) with research projects (Levine et al., 2011). Ellinas et al. (2019) found these same factors were also noted barriers that women ascribed to both promotion and leadership opportunities in AMCs.

The challenge for women to achieve sustainable leadership roles in academic medicine remains unresolved (Carr et al., 2018; Lautenberger and Dandar, 2020; Richter et al., 2020). A project committee of the Association of American Medical Colleges (AAMC) published a series of 15 recommendations aimed at building a comprehensive approach to increase the number of women leaders in academic medicine. These recommendations broadly entailed: “(1) developing and mentoring women faculty, residents, and students; (2) improving pathways to leadership; and (3) fostering readiness to change” (Association of American Medical Colleges, 1996, p. 807). A subsequent AAMC implementation committee prioritized and refined these recommendations into a set of strategic opportunities and actionable implementations focused on “the development of women leaders as central to the long-term financial success of the medical center” (Bickel et al., 2002, p. 1045). Notwithstanding the strong emphasis from AAMC on professional development of the individual as a primary factor to help achieve gender equity in leadership roles, some academic medicine leaders actually argued it was the institutional culture that must change to accommodate more readily the unique and beneficial traits of women physician-scientists (Andrews, 2002).

Professional development and leadership programs have evolved within AMCs to prepare the next generation of leaders—both men and women—for managing the increasing complexities of contemporary health care. Formally evaluating program impact, however, remains elusive as the predominant method of assessment is by satisfaction survey (86%), and only a minority of programs use assessments of learning (38%) or assess the actual behavior change of individual participants (30%; Lucas et al., 2018). One notable national leadership program for women faculty at AMCs is the Hedwig van Ameringen Executive Leadership in Academic Medicine (ELAM) program headquartered at the Drexel University College of Medicine, which has demonstrated both increased self-perception among participants in several leadership constructs (McDade et al., 2004) and a beneficial impact on career progression (Dannels et al., 2008). Recognizing the duality of both enhancing the skills and confidence of women to bolster leadership opportunities and also mitigating the flight of women from academic medicine, Chang et al. (2016) found a significantly lower rate of attrition among women faculty who participate in a career development program vs. both men and women non-participants. Lucas et al. (2018) conclude from their survey of 94 responding AAMC member schools that few employ a formal definition of leadership, a leadership competency model, or a theoretical framework of leadership as the basis for their programming, relying instead on *ad hoc* combinations of case discussions, lectures, and guest speakers that lack organizational context or a relationship to leadership theory.

### Resonant Leadership

Literature on leadership theory is vast and complicated. Many leadership styles or approaches are readily used in medicine that include transformational, servant, transactional, situational, and charismatic leadership styles (Avolio and Yammarino, 2013; Huynh and Sweeny, 2013; Trastek et al., 2014). Generally, all these leadership styles, and many others, have a place in the context of healthcare management. However, when considering career development and growth into leadership positions for women in medicine, resonant leadership offers significant value as collaboration through relationship building is believed to be paramount (Cola, 2019).

Resonant leadership is an approach premised on two primary dimensions: (1) the engagement of others through a positive emotional tone; and (2) a degree of connection with others or being in sync with others (Boyatzis and McKee, 2005). A resonant leader is positive and in sync with others, and a dissonant leader may use a negative tone and remain out of sync with others. Common characteristics of resonant leaders include positivity, relational awareness, empathy, compassion, emotional intelligence, self-awareness, and trust. By comparison, dissonant leaders are negative, unpleasant, often burned out, aggressive, lack self-control, disconnected, and usually unaware or overwhelmed (Boyatzis et al., 2012). Resonant leadership allows for renewal through mindfulness, hope, and compassion (Boyatzis and McKee, 2005) which is often reported in the career journeys of emergent medical leaders (Quinn and White, 2019).

People are often unaware of the power of emotions and their perceived impact upon others. Great leaders can move us by igniting our own passion and bringing out the best in us (Goleman et al., 2002). However, resonant leadership is not always about strategy, vision, or developing powerful ideas for success. It is prefaced on emotions that physiologically fire before our cognitive abilities are activated in the brain (Jack et al., 2013). Navigating complex environments, such as those situated in a healthcare context, and managing the dynamics of career growth must be facilitated by strategically leveraging both emotions and relationships with others.

Resonant relationships are the result of successful navigation by professionals through intentional change theory (ICT). ICT is one possible approach to explain the dynamics experienced by physicians as they encounter numerous points of alignment transitioning from who they are to who they want to become. ICT is a five-stage theory of discovery that begins with contemplation of the ideal self (Who do I want to be?), compared to the real self (Who am I now?), and the identification of alignment and gaps bridging the two states. ICT moves individuals through the creation of a strategic learning agenda, and a series of discoveries based on experimentation help emergent leaders practice new behaviors commensurate to their respective roles. This active experimentation culminates in the creation of resonant relationships (Boyatzis, 2008).

### Social Cognitive Theory

The genesis of social cognitive theory builds upon earlier behaviorist ideas first conceived by Miller and Dollard (1945) and Skinner (1957) who argued for a causative association between stimulus and response. In the view of early behaviorists, however, formal learning is achieved through reinforcement and repetition, with rewards provided for desired responses, and rewards withheld for undesired responses (Skinner, 1974). Rotter (1954) is the first scholar to advance social learning theory—an integration of behaviorism with German gestalt psychology to assert that a combination of both the social environment and individual personality interact to create the potential for a behavioral response. Two of Rotter’s seminal contributions include the underlying forces of expectancy [i.e., “subjective probabilities varying in magnitude from zero to one … (about) which behavior-reinforcement sequences are likely to be experienced”; Rotter, 1982, p. 249] and reinforcement value [i.e., “subjective value placed on the external reward or incentive that is important … (as) determined by past experience and present cues” (p. 247)].

Bandura and Walters (1963) extended Rotter’s social learning theory by asserting how individuals *also* learn through observation and by modeling the competencies of others. Social cognitive theory eventually developed independently as a model to highlight the more complex and multi-level environment in which individuals engage in learning and behavior change (Bandura, 1986). Among its unique contributions are the assertions of observational learning toward building skills as often demonstrated through modeling and the recognition of association between high levels of self-efficacy (i.e., the perceived belief in one’s ability to successfully execute a behavior), motivation, and personal goal attainment (Bandura, 1988). Social cognitive theory also emphasizes the reciprocal determinism between individual behaviors, personal factors (such as mindset), and the environment (such as social context; Wood and Bandura, 1989).

Social cognitive theory suggests a multi-modal view of human agency by distinguishing between personal agency, proxy agency, and collective agency (Bandura, 2001). Personal agency means taking intentional control to predict and shape events to one’s liking despite fortuitous or unfortunate circumstances. Proxy agency, however, may be preferential when an individual requires the perceived social efficacy of a third-party, or when “they believe others can do it better, or they do not want to saddle themselves with the burdensome aspects that direct control entails” (p. 13). Recognizing that the interdependence of social and economic life requires coordinative dynamics, collective agency represents working with others to achieve desired objectives (Bandura, 1997).

### Social Network Theory

While social network theory began as a largely statistical study designed to explain connections between individuals in a chain (Travers and Milgram, 1969), it quickly evolved to explore the interactions of network members and the structure of those interactions (Wasserman and Faust, 1994). An individual accrues social capital by virtue of their position within a social network (Coleman, 1990; Portes, 1998; Lin, 2012), and unsurprisingly, centrality yields the most advantageous position (Bavelas, 1948, 1950; Leavitt, 1951). However, Granovetter (1973) demonstrates the strength of weak-tie relationships, suggesting that the time and emotional intensity required to build innumerable strong-tie relationships prevents the robust network of weaker-tie “bridges” that yield potentially greater value as links between pockets of stronger-tie subnetworks.

The core ideas of modern social network theory are nicely articulated by Kilduff and Brass (2010): (1) social relations; (2) embeddedness; (3) structural patterning; and (4) utility of network connections. The interdependence of individuals—their interactions and relationships in a social context—is a well-trodden area of research perhaps first formalized by Comte (1853) in his coinage of sociology. Embeddedness relates to the principle that individuals tend to remain engaged with their social networks over time, continually renewing and expanding relationships (Granovetter, 1985; Baker and Faulkner, 2017). Structural patterns emerge in social networks, defined by organizational elements such as clusters, boundaries, and cross-linkages (White et al., 1976). Finally, social utility (i.e., value) is created when members provide opportunities for others within their social network.

Burt (1992, 2000) introduces the concept of structural holes to describe the phenomenon where two individuals share a tie but are not connected to each other. For an individual at the confluence of a structural hole, the opportunity exists to adopt a strategic orientation (i.e., act as a bridge, a facilitator, or an ally) or a manipulative posturing (i.e., act as an exploiter, a manipulator, or in an egocentric capacity). Obstfeld (2005) found that individuals with both a high strategic orientation and highly accurate cognition of informal networks engaged more readily with innovation-related activities.

## Materials and Methods

This study is part of a comprehensive evaluation of the FLEX Leadership Development Program that uses a mixed-methods approach consisting of program data, surveys and semi-structured interviews. Applicants to FLEX are assessed based on readiness for leadership training as indicated by their leadership goals and a clarity of vision for their future direction. As of June 2020, 127 women faculty had participated in eight FLEX cohorts. The FLEX Program selects participants from women faculty at the CWRU School of Medicine, including those professionals with appointments at CWRU’s four affiliated medical institutions: Louis Stokes VA Medical Center, MetroHealth Medical Center, University Hospitals Cleveland Medical Center (UHCMC), and Cleveland Clinic Lerner College of Medicine (CCLCM) at Case Western Reserve University.

Purposive sampling was used to select participants for interviews who were representative of every year of the program, from 2012–2013 through the 2018–2019 FLEX cohorts. At least two participants and up to six participants from every FLEX class were interviewed. Of the five affiliated institutions, interviewees were from all five institutions with between two and eight from any one institution. Both academic rank and highest degree mirrored the demographics of the FLEX Program participants, with 12 Assistant Professors, nine Associate Professors, three Professors, and one other rank. Likewise, the majority of those interviewed held an MD degree (19 MDs), three held a PhD degree and the remaining three were master’s level degrees. None of the interviewees were on the tenure track, which is typical of participants. Cultural background reflected all FLEX participants, with 68% Caucasian and 20% Asian or Pacific Islander and less than 10% for any other cultural or racial group. Most interviewees had worked at CWRU between 6 and 10 years, slightly longer than all FLEX participants (Table 1).

**Table 1:** Summary of demographics of interviewed FLEX Program participants.

Academic rank	Interviews (*n* = 25)	
	#	%
Assistant professor	12	48%
Associate professor	9	36%
Professor	3	12%
Other	1	4%
**Institution**		
UHCM	7	28%
CCLCM	8	32%
CWRU SOM	2	8%
MHMC	5	20%
VAMC	3	12%
**Highest degree**		
MD	19	76%
PhD	3	12%
MS	1	4%
PsyD	1	4%
MSN	1	4%
**Tenure-track**		
No	25	100%
Yes	0	0%
**Tenured**		
No	25	100%
Yes	0	0%
**Cultural background**		
Caucasian	17	68%
Asian/Pacific Islander	5	20%
Hispanic/Latina	2	8%
Other	1	4%
**Years at CWRU**		
0–5	5	20%
6–10	12	48%
11–15	5	20%
16–20	1	4%
>20	2	8%

UHCMC, University Hospitals Cleveland Medical Center; CCLCM, Cleveland Clinic Lerner College of Medicine; CWRU SOM, Case Western Reserve University School of Medicine; MHMC, MetroHealth Medical Center; and VAMC, Louis Stokes VA Medical Center.

Semi-structured interviews with 25 graduates between 1 and 8 years after training allowed for *post hoc* reflection on the impact and outcomes of personal leadership development in the months or years after FLEX participation. Interviews spanned all seven cohorts and breadth in academic rank, using eight core questions with probes designed to illuminate personal narratives using a constructivist grounded theory approach (Charmaz, 2014). We conducted a two-stage open- and axial-coding analysis (Saldaña, 2016) of collected data to address the three key research questions. The interview protocol was developed using the evaluation logic model for the FLEX Program to learn about individual participants’ personal outcomes (See Supplementary Material). Open-ended questions were used to elicit participant lived experiences during and after the program. Follow-up questions sought to obtain narratives regarding participant changes and progress. Some questions were designed to provide necessary feedback to help improve the program. The interview protocol is shown below in Table 2.

**Table 2:** FLEX interview protocol.

1.	Tell me what your experience has been with the Flex program? What do you think about the amount of training? How was this a transformational experience for you? Please describe your personal transformation experiences. What differences did others notice? What comments did they make about your leadership skills or your executive presence skills? How has your professional network changed after FLEX?
2.	Describe your progress to date, on your leadership vision? How has your leadership vision changed since you were in the FLEX Program? Where are you in the process?
3.	Tell me about a time when the FLEX program helped you complete, achieve, or manage something you were dealing with?
4.	Tell me about a time when you felt the FLEX program exceeded your expectations regarding achieving a goal that you were interested in accomplishing?
5.	Tell me about a time when you felt the FLEX program fell short of helping you complete, achieve, or manage something you were dealing with?
6.	If you were asked to help the FLEX leadership design a future iteration of the FLEX program what might that look like—What would you keep, what would you delete, and what new directions would you recommend?
7.	Executive coaching What was your expectation of what the coaching would be before you did it? How did that change after you met with the coach? What did you take away from your coaching experience? How did it inform your path to leadership? How many coaching sessions did you attend? Did you feel prepared? What was beneficial? What would improve the coaching sessions?
8.	What support is needed for FLEX Graduates after FLEX?

## Results

**Figure 1:**
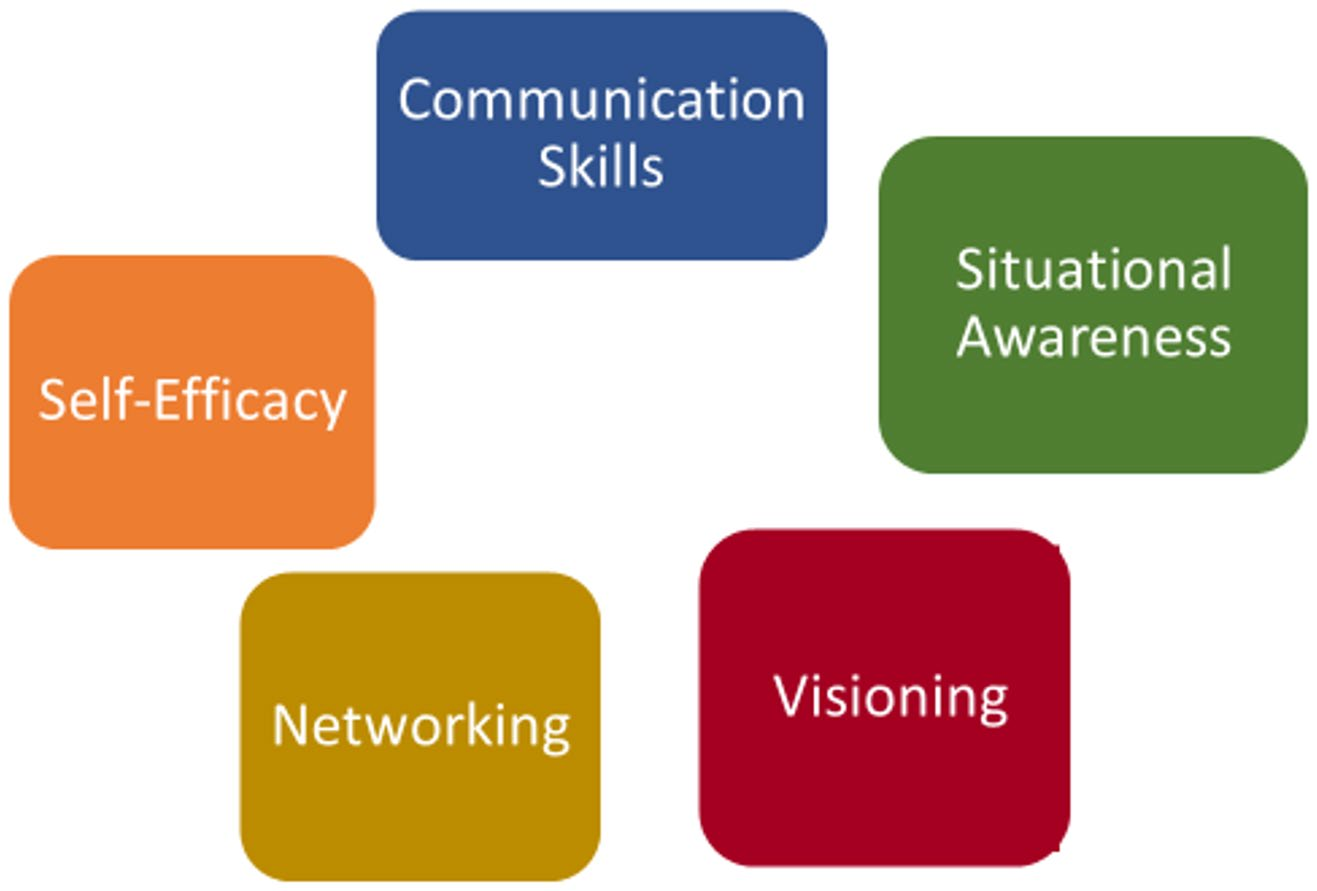
Overarching themes that influenced behavior change.

From the qualitative analysis of the interviews, the factors that facilitated behavior change following participation in the FLEX Program revealed emergence of five overarching themes: (1) Communication Skills; (2) Self-Efficacy; (3) Networking; (4) Situational Awareness; and (5) Visioning (See Figure 1).

### Communication Skills

**Figure 2:**
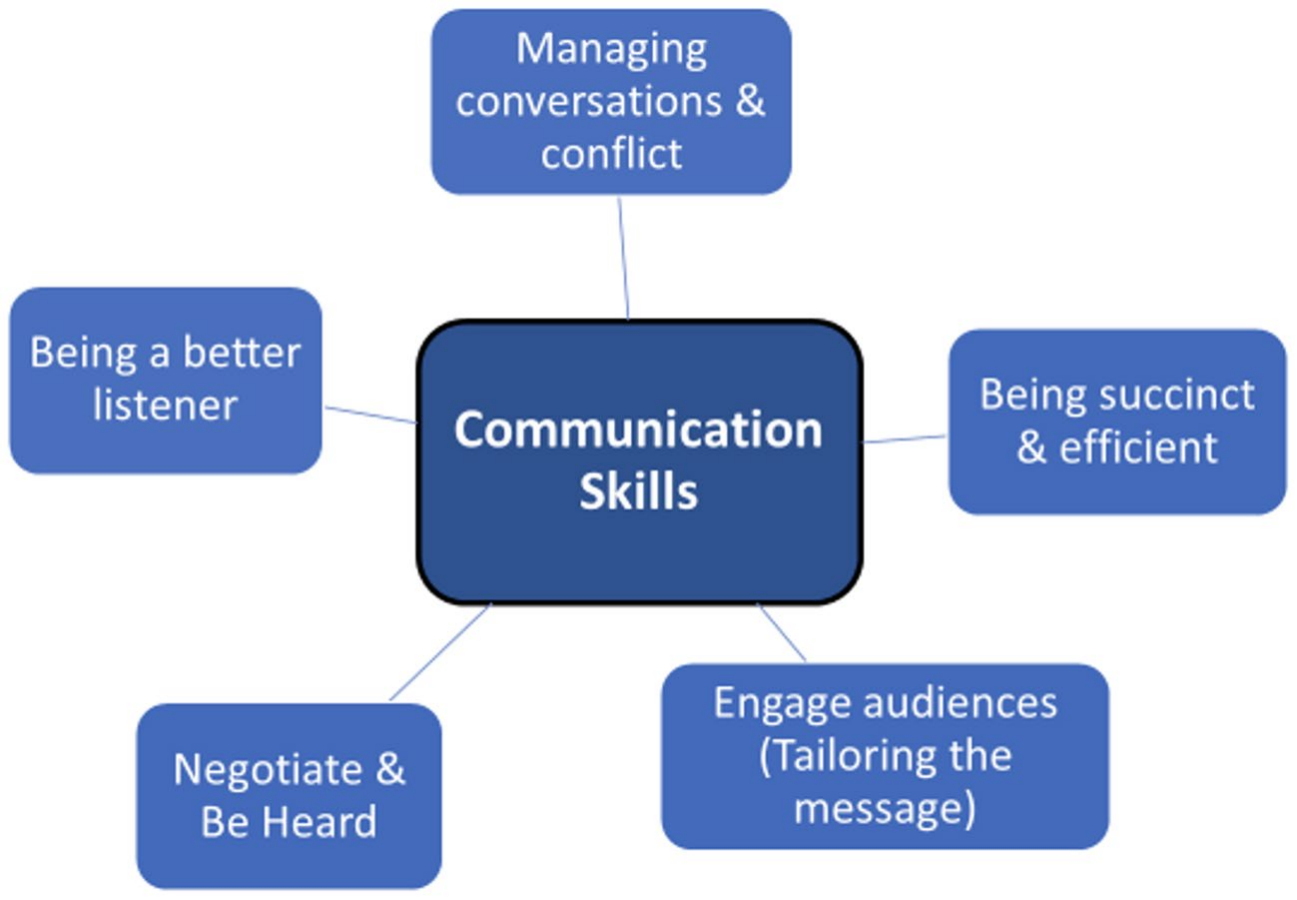
Communication skills.

Skills in several different methods of communication and learning about and applying different communication styles were critical in providing FLEX participants with strategies to move forward in their leadership development. The main communications themes were as follows: (1) Engaging audiences (Tailoring the message); (2) Negotiation strategies and being heard; (3) Being a better listener; (4) Managing conversations and conflict; and (5) Being succinct & efficient (See Figure 2).

#### Engaging Audiences and Tailoring the Message

A key takeaway for FLEX graduates was that the communications and executive presence training really facilitated improvements in communication and public speaking. They learned to analyze who their audience would be ahead of time and then reframe how they addressed them to appeal directly to that specific audience. Several participants said they developed their ability to have a conversation with their audience, such that listeners engaged more. This helped FLEX members overcome nervousness and uncertainty and gain confidence in their speaking skills. As a result, they received more invitations to give presentations. Several notable examples from participants included the following:

*“… [FLEX] helped me reframe how I gave talks and being more engaged with my audience. Feeling more like a conversation with them, rather than talking to them…” (FE13)*.

*“I feel much more comfortable now. I spend a lot more time trying to identify my audience. And be cognizant of making sure I’m speaking to the audience instead of just what I think they need to know…” (FE17)*.

*“When I look back on what I took away from [FLEX], it is really how to present myself in all different situations—whether I’m in a teaching situation, an academic presentation, or when I’m sitting at the table with leadership.” (FE61)*.

#### Negotiate, Ask for What They Needed, and Be Heard

FLEX members commented that the improved communication skills helped them to ask for what they needed and to feel like they are being heard. Better communication skills allowed them to clearly articulate their ideas and even sometimes spilled over into their personal lives as well, where partners commented favorably on their newfound skills. Developing an “elevator speech” is just one example where FLEX members learned to ask for or negotiate for what they needed.

*“… what FLEX allowed me to do was present it in a way that would be heard and be able to articulate my ideas in a way that I could be more compatible with the mission of the [organization].” (FE71)*.

*“I am able to communicate better. I can reach out to people better… I’m connecting with people in LinkedIn with an interest and then I’m asking … previously I used to be very hesitant at asking people for help. Now I’m asking for help… That’s something that I learned from FLEX.” (FE79)*.

#### Being a Better Listener

FLEX participants described the many interpersonal improvements that they observed with their newfound communications skills. They were better listeners, especially to alternative or dissenting opinions. This helped them develop into better leaders who could factor in several different peoples’ input on a matter. FLEX graduates began to appreciate how the different interpersonal styles could bring new perspectives and their newfound skills were even beneficial in their personal lives.

*“I also really try to be a good empathetic listener, and to make sure that I incorporate that into my leadership style to make sure that there’s adequate time for intentional listening.” (FE100)*.

*“I would say the transformation that I had afterward was being more thoughtful about my communications with others, not only in emails but also in thinking ahead, about planning meetings or even communicating what may be going on within a program I’m running. I found that to be very helpful because I think it made me more aware of other people’s interpersonal styles and that they may not work with ambiguity as well as I do…” (FE66)*.

### Managing Conversations and Preventing Conflict

FLEX members learned ways to deal with difficult conversations and how to manage people with challenging personalities. They also learned more about themselves and that they had gained skills that allowed them to be firm but also be true to their own personality. Sometimes the skills were applied immediately rather than saved for some future position.

*“[A colleague] wanted to take it in a different direction. There were just too many things that had to be weighed together. I asked to speak to him vs. the old me would have thought that avoiding that conversation may have been the way to go. The “FLEX me” thought that if there is an issue, I should deal with it early before it gets to be unmanageable.” (FE15)*.

*“I think FLEX reveals things that I did not know that I was able to do… For me it’s very difficult to tell people…this is not working…I learned that I can be still be nice … I think it gave me the tools to do that. So, I … thought at the beginning [of FLEX] that I was not able to do that, and now I feel empowered to do it.” (FE33)*.

Some respondents remarked about how improvements in interpersonal skills helped them outwardly demonstrate a change in the way they engage with particular situations, such as running meetings, leading coworkers and navigating challenging and complex situations.

*“I do think throughout the past couple years I’ve had to manage difficult interpersonal situations with workplace and a few of the exercises and techniques that we went through in the FLEX programs have definitely come in handy to help me…diagnose the situation and come up with a set of strategies to try.” (FE95)*.

*“We do an 8-h meeting … a business meeting that I run, and I have never done anything like that before… [FLEX] was transformational in just giving me the confidence to be able to do the work and run a meeting well. I think it was important to learn the skills that I needed to have for that purpose.” (FE13)*.

#### Being Succinct and Efficient

FLEX members noticed that they were more efficient at running meetings. Some said they were more thoughtful and better organized when preparing to speak. Likewise, improved presentations led to FLEX members receiving more invitations to speak both regionally and nationally.

*“A colleague came to me and said: ‘… I love to listen to you because your arguments are always so decisive and to the point and you present yourself so well.’ That was toward the end of the FLEX program. I had worked on that (with coaching) for more than a year but the FLEX program truly made the major difference. Thus, this was somewhat intentional, but it was surprising how applying what I had learned in the program made a difference so quickly.” (FE36)*.

*“I had to give a 10-min presentation of the whole program three times. I did it first when I was not in FLEX. Then the second time, when I presented, I felt so in control of my presentation skills without knowing… The [top leader] approached me and said, ‘[FE33], the content is always great, but your presentation skills—from the way you are talking and how you presented the content—was amazing.’ It was like day and night!” (FE33)*.

*“I ended up being the PI for the [institute]. That is taking a lot of work, and it’s requiring a lot of skill to manage communication across different departments, to manage navigating things … having complex conversations, and giving people clear instructions on what I need them to do so that we make progress…. The skills that I thought I would learn from FLEX and apply in the future when I have that [proposed center] put together, I ended up using them now to get the [institute] together.” (FE15)*.

### Self-Efficacy

**Figure 3:**
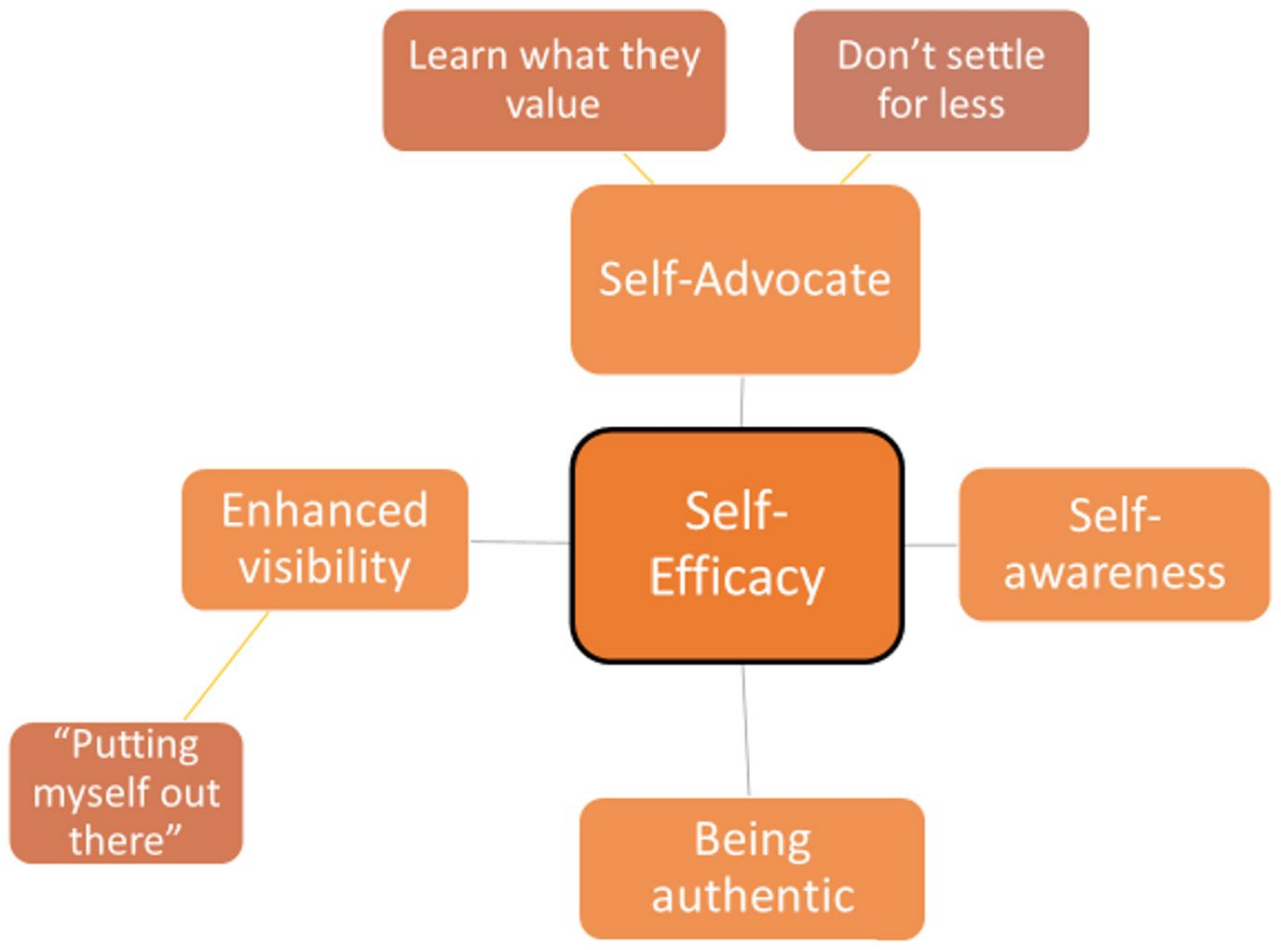
Self-efficacy.

During and after the FLEX Program ended, participants developed and increased their sense of self-efficacy and even confidence. However, the emergent finding seemed to fit best with the conceptualization of self-efficacy, which encompassed several themes: (1) Self-awareness; (2) Authenticity; (3) Enhanced visibility; and (4) Self-advocacy (See Figure 3).

#### Self-Awareness and Self-Confidence

FLEX members were able to see how they fit into the workplace and how they could modify their behavior to deal with particular situations. Their self-awareness also helped them to re-interpret other individuals’ words or actions with a different perspective.

*“I think I’ve definitely grown in those ways, that I do not know if I would have otherwise because I would not have had … any reason for self-reflection.” (FE48)*.

*“[FLEX] was very beneficial for me. It was really interesting to see [my colleagues], because it really gave me more insight into how [we] work together, and how I needed to adjust or to flex … to optimize our relationship in terms of how we work best.” (FE28)*.

The inward realization often leads to outward behavior changes. Occasionally, respondents remark both about how improvements in interpersonal skills help them inwardly evaluate their self-awareness and outwardly demonstrate a change in the way they engage with certain situations. Sometimes, this gave them a sense of belonging.

*“I had gotten these grievances that, ‘Oh, not everybody loved working with me’ … Initially, pre-FLEX, I was like, ‘It’s just all sexism. Everyone just hates me because I’m female’ …One of the things that [FLEX] helped me do is [reflect], ‘Okay. All of that may be true, but still, you have to manage how you are being perceived.’ [FLEX] gave me specific strategies, and that really helped… me identify my personality and interpersonal weaknesses in a professional environment. I was able to learn and grow from that.” (FE63)*.

*“I definitely think FLEX gave me the skills to improve in many areas to present myself in a more professional way, to be more confident…what’s been transformational is giving me a sense that I can have a seat at the table and it’s okay.” (FE102)*.

#### Authenticity

FLEX gave many of its participants the confidence to be themselves, to trust their own decisions and to present themselves authentically to the outside world. One FLEX graduate had a very difficult time making a decision to leave or not to leave her institution, but FLEX helped her remain true to herself.

*“[FLEX] gave me the self-confidence to believe that I actually did not have to ask for what I needed. …I feel like at that point in my career, FLEX … helped me to feel a little bit more peaceful… I’m going to be okay because I have something good to offer. If one school does not want me, then another school will…I feel like FLEX helped me to bring the decision more into my court, like it’s my decision, and I have the confidence to understand … that …I do not have to let somebody else make that decision for me, or I do not have to be afraid to go someplace else….It was almost…the most difficult decision I think I’ve ever had to make was whether to stay here or to leave, but I also felt great peace in making that decision. I think that the FLEX Program was so perfectly timed for me because it gave me that self-awareness, self-confidence that I needed to get through that period….I feel like it [FLEX] helped me, but in a way where I could stay true to myself.” (FE72)*.

*“I have learned how to be open to and trust my fellow FLEX colleagues. They have been one of the greatest strengths of the program. I learned to trust in myself, my voice, and my potential. This was been a career-changing opportunity.” (FE25)*.

#### Enhanced Professional Visibility

FLEX graduates reported achieving both personal and professional growth by drawing upon peer networks to proactively seek new leadership opportunities. They made themselves more visible by putting themselves out there and entering their name for leadership positions.

*“I think the other thing …is I’ve been working on getting involved in some leadership activities in other national organizations. I’ve been involved with one through the [national organization] as a [committee] co-chair… But now I’m putting myself out there more to be nominated, to be on boards for different organizations and things…that I had not done before.” (FE66)*.

*“…One of the things I gained was in order not to feel stagnant in what you are doing you have to keep looking for ways to grow or to push yourself. I do think I look for these opportunities because they are interesting and they do help me… In my job, I see myself as having moved from the person who’s in charge of the nuts and bolts of things to the person who’s a guide for the trainees, so I need all the tools I can get to be a good guide.” (FE84)*.

*“Now I’m asking for help. … I was thinking, ‘Someone will help me out. Someone will do it for me.’ That’s not the way the system works. You have to present yourself into the system. That’s something that I learned from FLEX.” (FE79)*.

Conversations in FLEX helped some participants identify their goals and this prompted some to consider going for leaderships roles that they might not have considered otherwise.

*“[The other FLEX members felt this was] …going to be transformational for me, in terms of more contacts and more work and really to move a career forward.” (FE62)*.

#### Self-Advocacy

FLEX members learned to recognize their value and then they can speak up for themselves by making it clear what value they bring to the situation and not settling for less. Likewise, they look for the best deal where everyone benefits (e.g., a win-win).

*“I felt like that was a huge victory for me in which I never had gotten it… in the previous 5 years… I feel like the skill with FLEX, … it certainly gave me more knowledge and perspective, and confidence in being able to present myself and being secure in that, and really being okay with walking away from it too. Because if they were not going to pay me for what I was worth, then I wasn’t going to do it. My time is really valuable, to make the people that I was negotiating with understand that… I think they realized … I was able to present myself concisely and show what I’ve really done within my own division, and how I can achieve that in other places throughout the system.” (FE30)*.

*“The FLEX Program did a couple of things. I think it … helped me to understand a little bit more about how to…professionally present myself, I think it just helped me through the … interview processes, but I also think it increased my confidence a bit, and I think … it helped me to at least think about asking for what I needed.” (FE72)*.

### Networking

**Figure 4:**
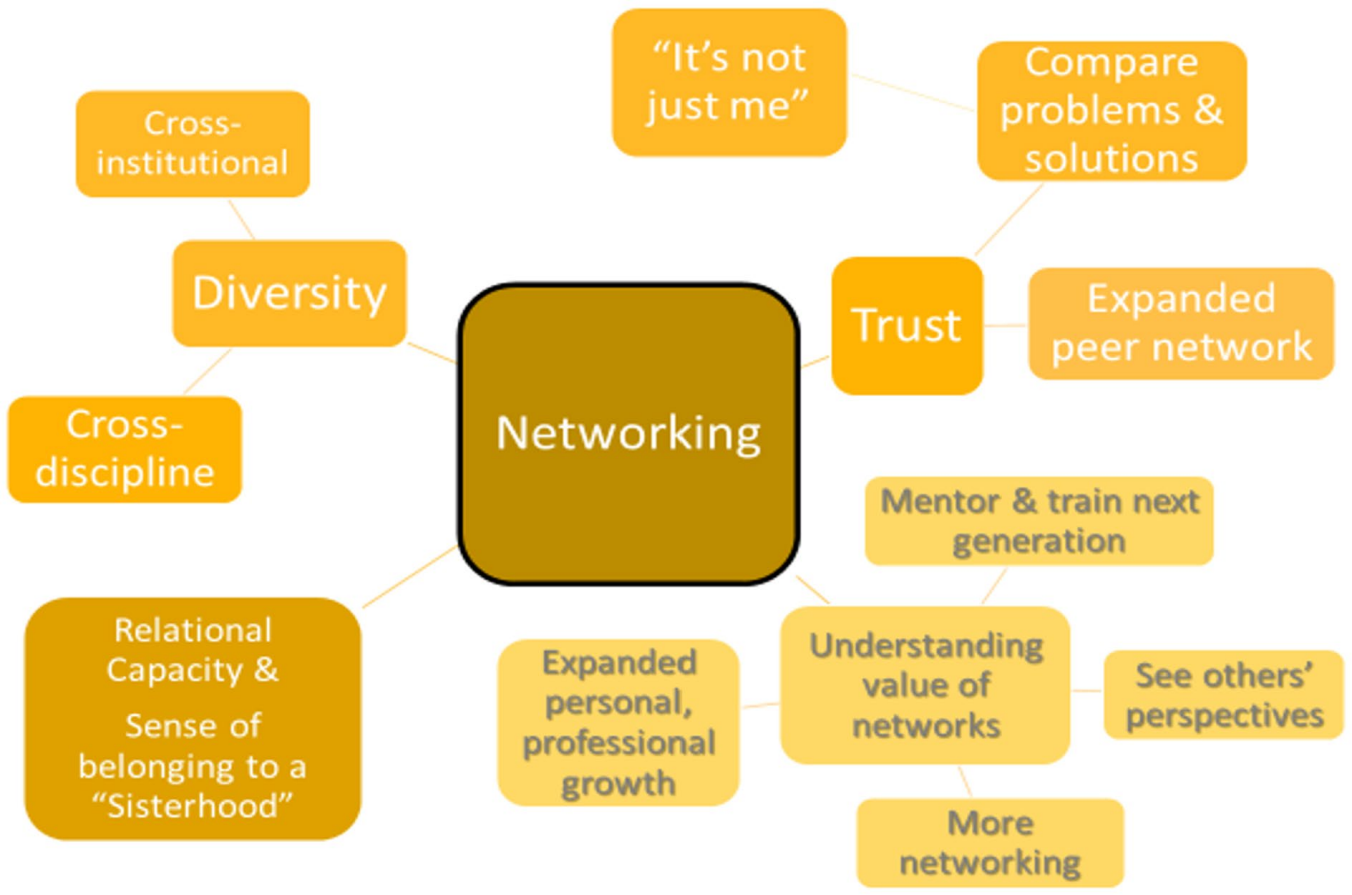
Networking.

The director of the FLEX program referred to the networking aspects of FLEX as the “hidden curriculum,” because she had observed that there were significant benefits from simply going through the program together with other professional women. Those benefits fell into several related themes: (1) Diversity of the participants; (2) Trust and Transparency; (3) Understanding the value of networking and mentoring; and (4) Increased relational capacity (a.k.a. the FLEX “Sisterhood”; See Figure 4).

#### Diversity of the Participants

Most FLEX participants said they felt the relationships they made with fellow members of their cohort were critical to the success of the program for them as an individual. For many participants, the diversity of the individuals in their class was highly valued because they were able to develop relationships across disciplines, across institutions and with women who were at various stages of their careers.

*“Being able to talk and visit a cohort of women who are in different stages of leadership, and listening to their struggles and learning from their model helped to conceptualize my philosophy of leadership.” (FE34)*.

*“The part that had the most enduring impact was that I met people at different stages in their careers and developed relationships and they were people who I otherwise would not have interacted with. For example, [women physician] and she’s pretty senior. I never would have come across her, but she became one of my greatest mentors…” (FE63)*.

*“I think the biggest strength was probably the cohort of individuals …. I think outside of my field, I do not necessarily get to interact with a lot of faculty in the [medical school]. I think that was really one of the benefits to meet this group of women who are all trying to further their career.” (FE23)*.

FLEX fostered cross-institutional relationships that would not have happened otherwise. The diversity of the relationships meant participants could learn new ways to tackle similar challenges.

*“I also think having connections with people who aren’t necessarily in my discipline has been helpful to get other perspectives” (FE66)*.

*“So the formal teaching was great, but then the in-between time, the break time conversations, with people from other institutions, to see what was similar, what was different, what would work in a different environment or with a different recipient when you were given the message. I think that was really valuable.” (FE62)*.

#### Trust and “It’s Not Just Me”

FLEX provided a supportive, trusting environment for participants to feel that they were not alone in dealing with difficult situations in work environments. It also provided some alternative solutions to challenges that participants might not have considered.

*“They’re different in that it’s like I feel like I can trust those people. …So, I guess there’s a trust that I feel, and also… we do not have to develop that collaborative relationship, it’s already there. …I know if I need you, you are going to be there for me, and I’m going to be there for you.” (FE72)*.

*“I have learned how to be open to and trust my fellow FLEX colleagues. They have been one of the greatest strengths of the program.” (FE25)*.

*“One of the other things I learned in the program was about work-life balance… by talking to the other participants in the group. Understanding what worked and not worked for them has been a tremendous help.” (FE89)*.

The FLEX program helped participants realize they were not the only ones experiencing these challenges and this group support was invaluable. FLEX resulted in relationships with underlying trust which fostered advocacy for fellow FLEX members.

*“The knowledge of others facing similar challenges has greatly helped me.” (FE46)*.

*“It was really important to see that it wasn’t just me. I wasn’t the only one who was having some challenges in terms of acclimating into a new role or getting comfortable in kind of a leadership position.” (FE28)*.

*“It’s somewhat nice to know that even though we have different jobs and different job titles, we experience a lot of similar challenges that we can learn to solve from each other’s experience.” (FE95)*.

#### Understanding the Value of Networking

Learning the value of networking changed FLEX participants’ behavior regarding future networking so that now they actively reach out more and seek new relationships, are more likely to mentor trainees or to see other perspectives. Participants felt that the FLEX members provided a potential networking resource for the future. After FLEX, they saw the value in fostering stronger or more focused professional relationships and they had developed the confidence to do that. They were now more willing to reach out to each other.

*“I came back … and started a women’s network here. It … inspired me to do other things, and so I know people within my institution a lot better.” (FE71)*.

*“I think we have now our networking … we work faster, we share things together. And we all feel that it was transformational. I do not know if everyone … but in general we are very committed to continue the relationship between one each other.” (FE33)*.

*“I do feel like I am more willing to go out of my way to reach out to people, not within the FLEX network but just in general, in my own professional arena. I do think it gave me the confidence to expand my network or to make my own networking opportunity.” (FE95)*.

Some relationships transformed into mentoring relationships. FLEX participants found it easier to make relationships, but not necessarily specific to FLEX members. FLEX helped them improve at reaching out for help from others and valuing those connections more. In some situations, this boosted their trajectory toward positions of leadership. Thus, FLEX members learned the value of mentoring and networks and now train others in networking.

*“I learned not only how to enhance my network myself, but I also learned more about the value of helping others enhance their networks and how to teach and train around that.” (FE64)*.

*“…what was transformational … and I’ll keep going back to this … was the interaction with the other people in the group and the lasting mentorship relationship that I was able to have.” (FE63)*.

*“I think I used FLEX to… reprioritize what I wanted to do with work. A byproduct of it was I have been able to mentor some of the junior faculty here and in the process, give them the opportunities that I did not necessarily have time for or interest for, but give them the opportunity if they did.” (FE71)*.

*“…one of the tools that I probably used the most—and I teach people the most—is the PREPO*^1^ *[method]… I use it to help teach [trainees] how to respond on the spot…I found that not only it helps me but I’ve been able to use it as a teaching tool to help mentor and foster other potential leaders…” (FE66)*.

#### Increased Relational Capacity

FLEX graduates developed deep and meaningful interpersonal relationships fostered by showing each other vulnerability and developing trust. Leveraging this “FLEX sisterhood” network emerged as the most salient benefit that graduates reported from completing the 7-month program. The “hidden curriculum” in the FLEX Program is the relationships that the participants form with each other.

*“It’s … a little bit self-help group-ish, where you find value in knowing just that other people are going through something similar.”(FE28)*.

*“…I feel very close with the group. We’re like sisters in that regard. I do not see enough of them often enough, but it’s always… a warm feeling when you bump into anybody. And when you see a success from one of the group, it feels wonderful. So, I do think… this group will stay with me forever.” (FE17)*.

*[To a fellow FLEX graduate] “Oh yeah, you did FLEX. You know what it takes. You know what is involved when you do a deep dive into yourself and try to figure some things out…” and “…we have this kind of little safety net…It gave me a sisterhood of people that I still call when I have career things to decide, which is awesome.” (FE80)*.

This close-knit sisterhood develops over time and encompasses trust and psychological safety. Participants realize that other women are going through similar experiences and they become more comfortable sharing their challenges. Some of the relationships develop into long-lasting friendships and others develop into strong collegial ties and mutual mentoring.

*“…it seems like we are already seeing some tremendous successes and I get just energized and excited about hearing and seeing other people hit these milestones or have these accomplishments. It kind of motivates me further too…. it’s so cool to be able to connect these people and … see us put some of these things into action and also having that familiarity with those other individuals in the room because it’s a new role for all of us… we can be each other’s coach and support.”(FE35)*.

*“…it’s a great sounding board for validation, but also, it kind of makes you hold yourself a little more accountable.” (FE35)*.

*“I also utilized networking tips discussed during our FLEX lunch sessions to introduce myself to thought leaders at regional conferences. This led to further networking and an unexpected invitation to participate on a national committee.” (FE43)*.

### Increased Situational Awareness

**Figure 5:**
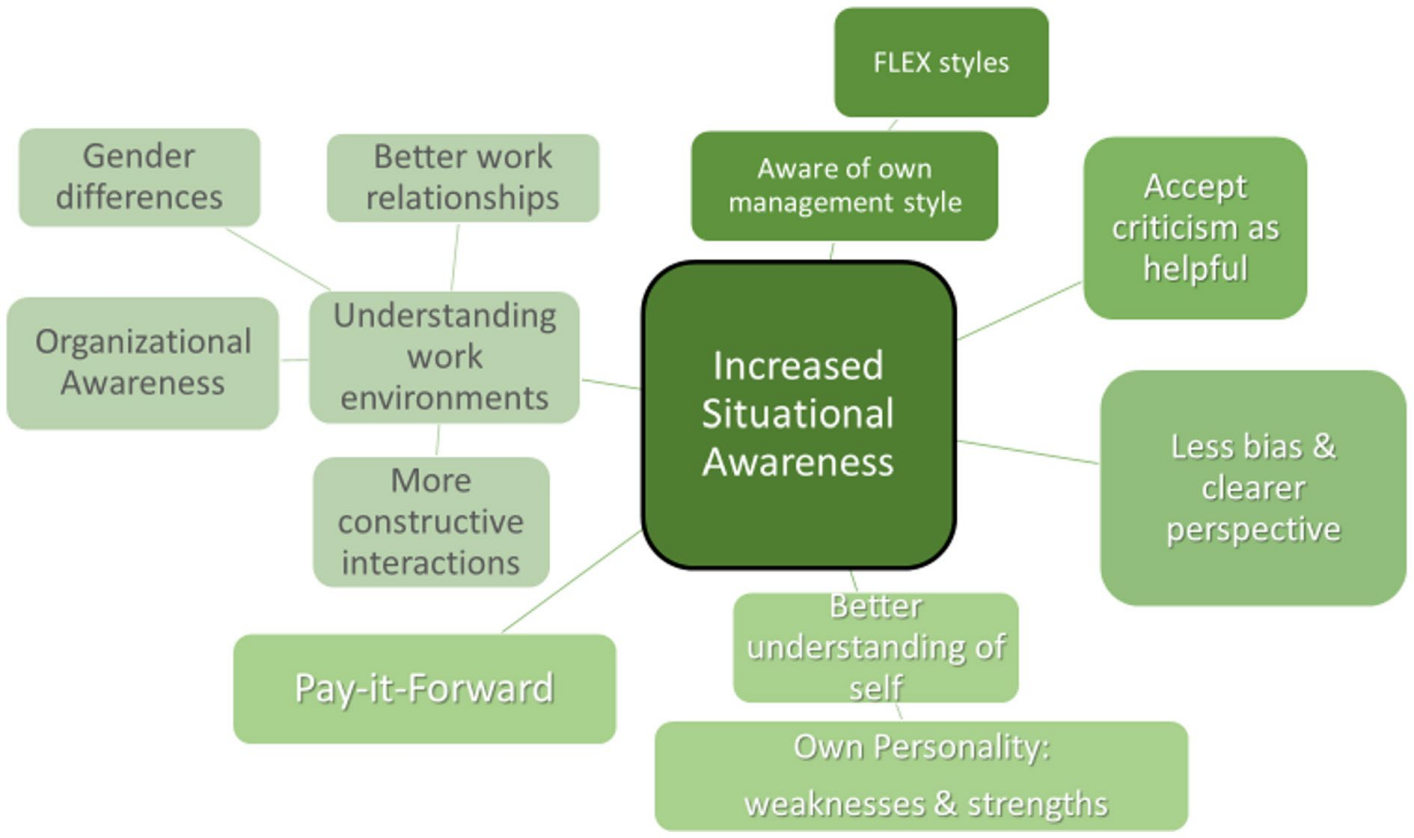
Increased situational awareness.

Understanding the work environment is more than just understanding the climate, it is realizing where they fit best into the environment and having the confidence to recognize what they are good at doing. A better understanding of the work environment was critical for many FLEX participants in helping them develop the skills they had learned and in navigating the sometimes-difficult work environments. The main themes that emerged were the following: (1) Understanding the work environment (work relationships, gender disparities, and more organizational awareness); (2) Becoming aware of their own management style and how to “flex” styles; (3) Accepting criticism; (4) Having a clearer perspective and showing less bias; (5) Developing a better understanding of self; and (6) Pay-it-Forward mentality (See Figure 5).

#### Understanding the Work Environment: Gender Differences

A big part of understanding work environments is becoming more aware of the where a person fits into the hierarchy. Sometimes, that means learning to understand how gender can affect the work environment. Another part is gaining the confidence to manage groups in terms of communication and delegation. In one case, understanding the work environment led to improving the relationship with a supervisor and working more constructively going forward. FLEX helped make women comfortable with making big decisions knowing that they are heading in the right direction for their career. Situational awareness at work helped FLEX members advocate for others. One FLEX graduate felt strongly that getting into top leadership positions depends on who you know and having influential people sponsor you. She felt that it is also a matter of understanding what is expected and women do not always seem to have that political awareness of how to signal that they are serious about a leadership position. In one case, this meant speaking candidly to leadership and having the confidence to point out inequalities in the workplace that may be perpetuating gender stereotypes.

*“In academic medicine, … when you look at the balance between men and women and you see men going into higher and higher positions and women sort of falling out, I think that there is what I call a cultural epigenetics where a lot of times, women, just because they do not know that they can do something …or how to do it, they miss out on opportunities… I continue to be deeply troubled by the lack of women in leadership positions.” (FE63)*.

*“I liked the fact that [FLEX] wasn’t specifically career oriented, and we really talked a lot about the troubles and the struggles that we have as women trying to balance families and careers.” (FE30)*.

#### Organizational Awareness, Being Aware of Your Own Management Style and Learning to “Flex” Styles

FLEX graduates developed an ability to manage issues contextually through increased internal and external situational awareness. They learned more about their own management styles as well as how to “flex” or accommodate other’s styles.

*“…[FLEX] really gave me more insight into how [we] work together, and how specifically, … I needed to adjust or to flex, … to optimize our relationship in terms of how we work best.” (FE28)*.

*“I do not think I would have had as much support had I not done FLEX, …. I think FLEX was an important part of this readiness process of moving into a role of understanding a little bit more about leadership and management, and really kind of understanding a little bit more about yourself.” (FE28)*.

*“I became aware of my style of management more and became aware of how that could interact with other styles of management or perceiving things…. it just made me aware that this is something that could clash with others.” (FE11)*.

For some graduates, FLEX provided them with a better understanding of their own strengths. One FLEX graduate said that her rationale for applying to FLEX was that she started a new administrative leadership position and she wanted to learn skills to do a better job. She learned about what sort of members you want on a high functioning team as well as what sort of team member she was:

*“…one of the things that struck me … was how I knew exactly where I fit…But it really made me better able at articulating what my strengths are and what I could bring to a team.” (FE23)*.

One FLEX graduate said that she “felt a comradery with other FLEX graduates at her workplace, knowing that they all had been through an intense self-evaluation and self-realization process.” She felt that FLEX helped her and others to adapt and to address the question:

*“How do you find enough of your own self in order to really tackle some of these challenges, but to do it in a way that’s professional … that speaks to the type of person that you are, but also is appropriate for a workplace, but that also leads down the road to your continued professional development and helps others along too?” (FE28).*

#### Less Bias

FLEX participants said they learned how to see things more objectively and perhaps with less bias and a clearer perspective.

*“These aren’t people with whom I would naturally have clinical overlap, but they are still doing work and making progress in areas that … I just would not be exposed to. So … learning from their successes and their challenges and … seeing something outside of my own silo, I think is … very helpful to… see the bigger picture, get some perspectives from different things.” (FE35)*.

*“I think one of the great strengths of the FLEX group is that it’s not just everybody …in the same exact specialty, that there is …some strength that’s built by the different perspectives that people are going to offer.” (FE48)*.

*“I feel that I’m more thoughtful. I feel more mature after having gone through the FLEX course.” (FE102)*.

*“I am less afraid of conflict. I pause now and try to think through, ‘What are my go-to conflict management styles? What do I think are the other person’s? Is this a time that I should be trying something different than is the norm for me?’ Up until that point, I had usually been avoiding or competing, so I’ve definitely moved more toward collaboration … with my colleague peers. I’m generally more open-minded. It does not have to be my way. I might not agree with somebody else’s solution, but it’s okay to let it play out and see what happens. I do not have to control everything.” (FE84)*.

#### Pay-it-Forward

Some situations resulted in a “pay it forward” mindset where one FLEX member wanted the best result regardless of whether she received credit for it.

*“… I think FLEX helped me with that…is this your goal? Because if your goal is because you need some sort of personal glory, [or if] your goal is because you want a [great] program that looks like this. And if there’s key people that you know have talents that can better present this … it’s going to be better then, it does not necessarily have to be all mine and I think that’s probably transformational too …I think it’s maybe a mindset change.…you want to see a program that better delivers health care in this way, … what really needs to be a job goal or administration goal and FLEX really did help me personally … look at that and say yeah, like at the end, it does not matter.” (FE62)*.

### Visioning or Forward-Looking Personal Introspection

**Figure 6:**
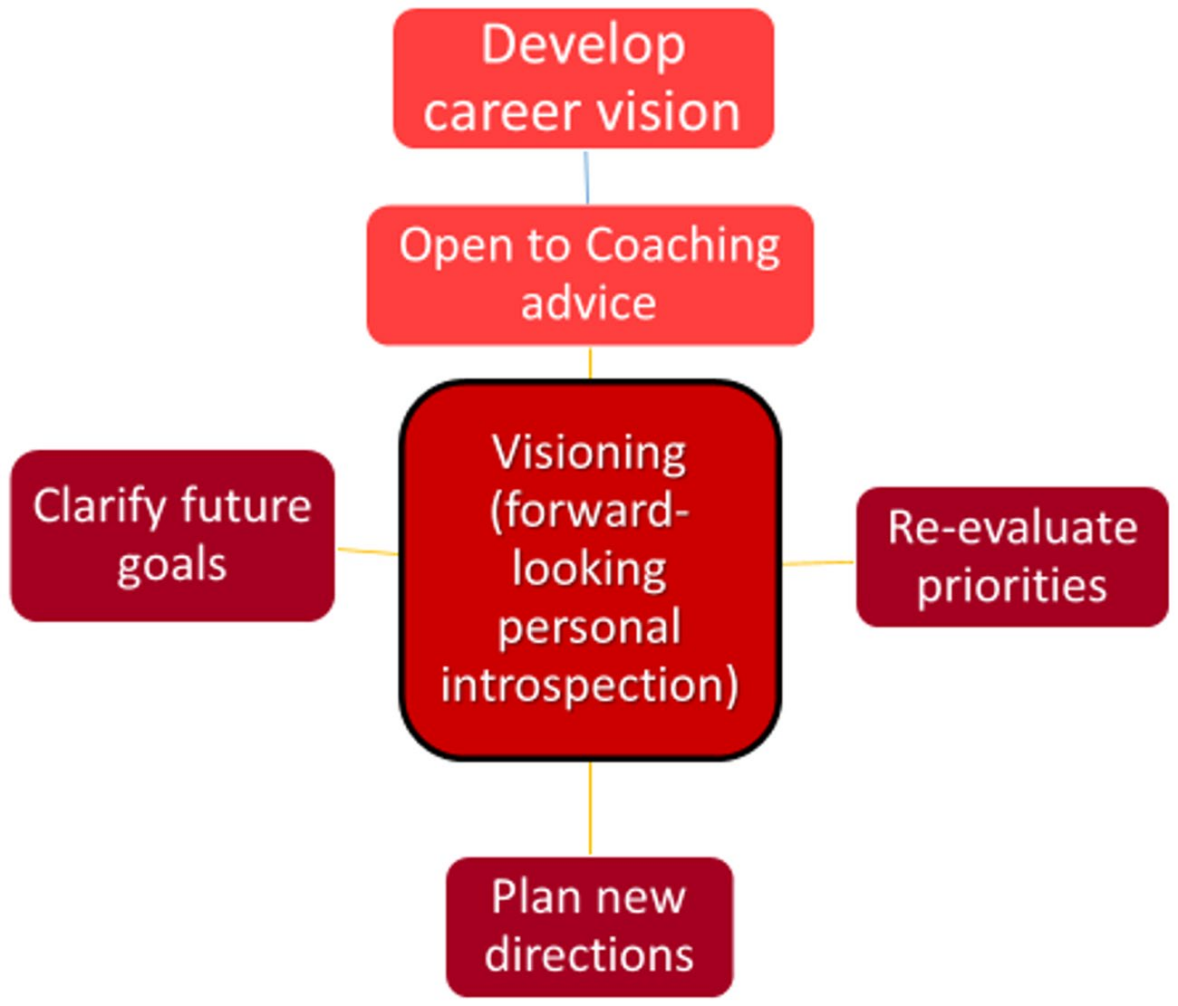
Visioning.

There is a demonstrable phenomenon of personal reflection among many FLEX participants where the concept of “visioning” leads to personal leadership growth. This is often, but not necessarily, coupled with professional leadership growth. The main visioning themes that presented in the data included: (1) Clarifying future goals; 2) Re-evaluating priorities; (3) Making time for forward-looking activities; and (4) Developing a career vision (See Figure 6).

#### Clarifying Future Goals

The coaching experience can have such a profound influence on individual behaviors, goal-resetting, and shared vision. Consider this FLEX graduate, still a practicing clinician, but focused on maintaining a stronger work-life balance:

*“I’ve always had a personal struggle with the “Should I want more?” I’ve liked and been motivated by external things in the past. …As I get older, I am more comfortable saying, “You know what? … You do not need that. You will not actually enjoy it for the most part, so why do it?” I do not know that it’s so much a result of FLEX as it is just me better understanding myself and my values. … Concurrent with completing [FLEX, I had a] readjustment of priorities. … It’s nice that people trust me to do things and think I do a good job, but I actually just really want to spend time with my family and not be working. And that’s okay, too. I do not have to be everything.” (FE84)*.

#### Re-Evaluating Priorities

For some FLEX participants, personal introspection and visioning can manifest as catalyzing profound life changes. To critically evaluate life’s priorities through the lens of self-fulfillment can be a humbling experience for professionals accustomed to “the grind.” Self-fulfillment can shift the individual reward orientation from extrinsic (or pro-social) to intrinsic motivating factors. Some participants said they acquired a vision along the way, or some saw themselves differently as a result and consequently their vision changed. Still others said that FLEX really helped them clarify their future goals. Some examples of FLEX members’ statements about their career vision:

*“Yeah, a clearer plan for sure. Yeah, it was a very idealistic plan, … you have to manage that idealistic plan with what you can do. But, I mean, [the coach] helped me visualize how much time I wanted to be doing clinical work and how much time I wanted to be doing my other endeavors.” (FE26)*.

*“I recall what I got out of it was starting this process of reevaluating my positions at work….which ones do I want to … become a leader in … which probably prompted me to start thinking about prioritizing all my roles.” (FE71)*.

This underscores the importance of developing an ideal vision and plan for attaining it. It might be possible to outline a “leadership trajectory” for early-career physician scientists, such that they can develop the tactical steps required to achieve their long-term vision, avoiding those activities that provide little intrinsic value and accept those activities that provide self-fulfillment.

*“After FLEX, I took [time off]. My expectations for myself—career goals and personal goals—needed some reshuffling … I stopped working. I went [abroad] for a month and volunteered … there, and then I traveled around for an extra couple of weeks…. I realized that I really do miss medicine and the connection that I have with the residents and my colleagues here, so I got back into doing some teaching….I used FLEX to reprioritize what I wanted to do with work. … I made my entry back into work doing only the things that I really enjoy. I pretty much weeded out all of the committees, projects, things that were not fulfilling to me.” (FE71)*.

#### Forward-Looking Activities and Planning New Directions

“Visioning” behavior is adopting the FLEX skill of making time for personal introspection: a scheduled and dedicated time to envision future plans and curate an ideal picture of what is fulfilling. For some FLEX participants, visioning is a regularly-scheduled meditative practice allowing time for personal introspection.

*“It was actually the last session where I figured …. I need to take time to do forward-looking activities. … So I did, I made the time… I just decided that the priority for that hour-and-a-half was going to be forward-looking activities … whether it’s pulling research articles or even if it’s just planning my meetings or sending emails, all of those things have to be in common with … future thinking. …Before FLEX, I would have wanted to do it and … never made the time.” (FE26)*.

*“I think that, unless you build that time in, you will not get it. You’ll just keep working, and doing your daily tasks.” (FE13)*.

#### Developing a Career Vision

FLEX graduates said that FLEX helped them clarify their career vision and move forward in ways they had not been able to do before FLEX. It also helped them gain confidence in their abilities, which opened up new possibilities that they had not considered for themselves. This led FLEX graduates to begin to ask for the things they needed and pursue leadership opportunities. Several FLEX graduates went on to develop professional career development programs at their own institutions.

*“What I’m trying to say here is that when we write that vision, we have one place where we want to go, but what ends up happening is the skills that we learn can take us in other directions that may be equally as important, if not more.” (FE15)*.

*“When I took the FLEX Program, my vision was to utilize the FLEX Program in my own vision of professional development, since I lead professional development within my department. So that kind of helped me import ideas and ways of doing things.” (FE11)*.

## Discussion

These results suggest that the enduring benefits of the FLEX Program include improved communication skills, expanded relational capacity (Cola and Wang, 2017) through networking, greater situational awareness, improved self-efficacy, and enhanced professional visibility. In addition, FLEX was a transformative experience when participants articulated their leadership vision and developed a plan to execute their goals toward attaining leadership roles. A significant barrier hindering behavior change is a lack of experience and insufficient time to practice those skills acquired from FLEX training sessions. Participants also report needing time to improve underdeveloped interpersonal skills, and they need to address time management issues to create opportunities for mastering new behaviors.

Improved communication skills were prominent among the factors that had the greatest effects on behavior change. The most significant reported communication skills developed by FLEX graduates include improving interpersonal skills, greater ability to manage conversations, and competence in presentation and public speaking. Verbal and non-verbal communication skills training helped FLEX members overcome nervousness and become more efficient at advocating clearly for themselves. Professional presence training helped FLEX members reframe how they address an audience, provided confidence in their speaking skills, and as a result, they received more invitations to give presentations. Improvements in interpersonal skills development helped graduates both inwardly evaluate their situational awareness and outwardly demonstrate a change in the way they engage with other people.

Building relationships and networks emerged as the most salient factor graduates reported from completing the FLEX program. The overall take-away for relationships and networking was that the diversity of members in each FLEX cohort was critical for developing relationships outside the members’ typically discipline-specific connections. The program fostered cross-institutional relationships that would not have happened otherwise. FLEX participants came to recognize and appreciate the value of networking so much more and they had developed the confidence to make new connections. The improved networks fell into three categories: (1) Better relationships in general, or participants found it easier to make relationships, but not necessarily specific to FLEX members; (2) FLEX now provided a potential network for the future. After FLEX, they saw the value in fostering stronger or more focused professional relationships and they had developed the confidence to do that. They were now more willing to reach out to each other; and (3) Strong bonds of trust with their fellow cohort created a sisterhood, where members realized others were experiencing the same issues and were going through the same challenges. This shared experience fostered trust and helped FLEX members see other people’s perspectives better. Knowing how valuable the networking was, it changed their behavior regarding networking so that now they actively reach out more and seek new relationships and are more likely to mentor trainees. FLEX graduates reported achieving both personal and professional growth by drawing upon peer networks to proactively seek new leadership opportunities. In some cases, these relationships led to increased teamwork and clinical/research collaborations.

With regards to personal and professional growth, one of the top reported outcomes was increased self-efficacy. Participants said the increased level of confidence allowed them to be better negotiators, develop valuable new collaborators, to reach out and depend on their newfound peer networks and to seek leadership opportunities that they would not have sought previously. Several participants commented that they are now better leaders because of their newfound confidence.

Improved situational awareness starts with increased self-awareness and then matures to a better understanding of one’s own management style and flexing styles and ends with evaluating priorities and clarifying goals, which sometimes means changing directions. The most frequently expressed type of personal growth was a better understanding of themselves. This included a better understanding of their own emotions and reactions to situations and an enhanced ability to deal with difficult personalities, situations and provide negative but constructive feedback. In several cases, the FLEX member’s improved understanding of themselves helped them to re-assess their work situation and make changes for the future that would take them in a new direction that was more consistent with their goals. This was supported by both interviews and independently by answers to end-of-year surveys. Primarily participants described understanding more about themselves as a person and this helped them gain confidence and develop stronger relationships. Graduates could imagine themselves in leadership positions. Some participants described personal growth in areas outside of their work and professional lives.

FLEX provided a supportive environment for participants to feel that they were not alone in dealing with difficult situations at work. A large part of understanding work environments is becoming more aware of the hierarchy and where a person fits into that system. A prominent theme that arose from FLEX graduates was how to negotiate and make clear their own value and not settle for less. A better understanding of the work environment also led to a “pay it forward” mindset where FLEX graduates wanted the best result regardless of whether they received credit for it. Most FLEX graduates currently mentor or plan to mentor other women in academic medicine.

Important strategies acquired by participants included setting aside time for goal planning through personal introspection and visioning. A personal leadership vision often emerged from executive coaching sessions in which participants construct a plan for achieving their leadership goals. However, not all participants engaged fully in the coaching process, resulting in mixed sentiments about its potential benefits. FLEX graduates overwhelmingly desire ongoing support and skills reinforcement to help sustain the benefits. FLEX graduates report achieving both personal (e.g., moving toward an ideal vision) and professional leadership growth.

### Factor Interactions That Help Influence Behavior Change

**Figure 7:**
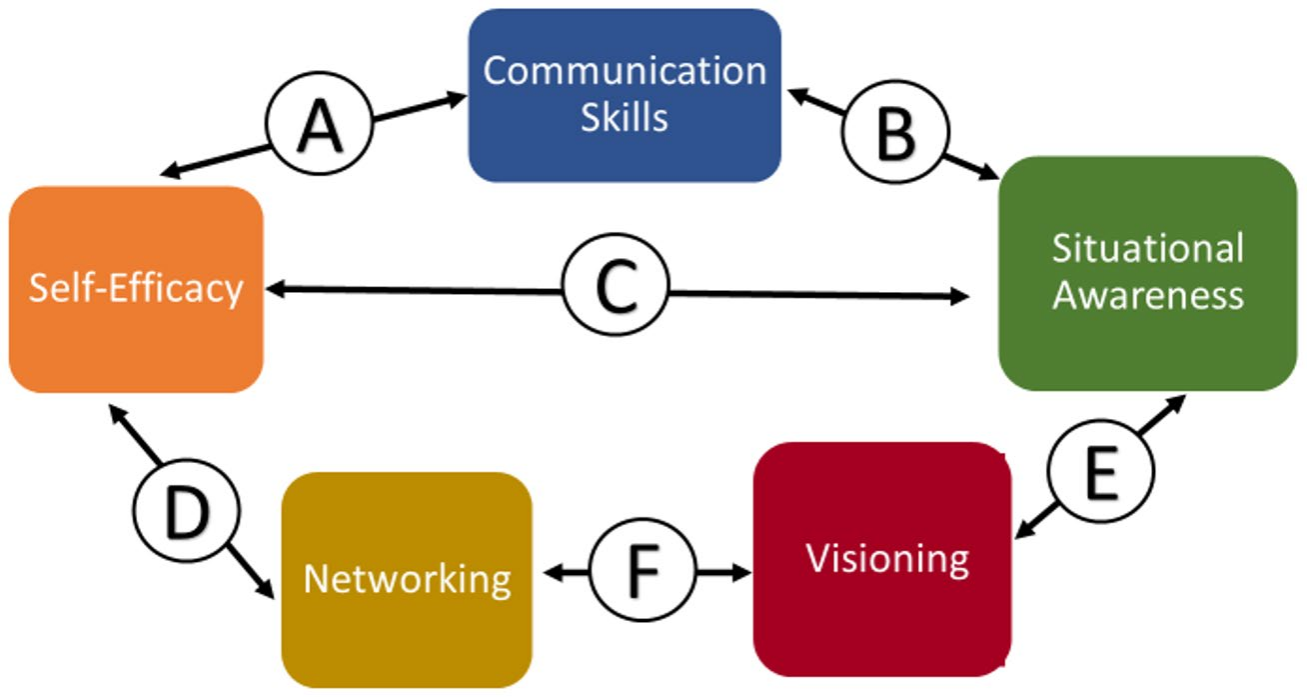
The interactions between factors that influenced behavior change in leadership development.

Significant benefits resulted from the behavior changes induced by FLEX program. The new professional presence skills acquired along with greater self-efficacy and improved situational awareness all interacted to provide significant benefits to the FLEX graduates. Although all these changes were happening simultaneously, in the following section they are broken down separately to facilitate understanding. The results showed significant interactions between the various factors that influenced behavior change (Figure 7). Each interaction is labeled with a letter A–F, and is described below.

#### FLEX Participants’ Self-Confidence in Their Speaking Abilities Helped Them Achieve Greater Visibility and They Received More Speaking Invitations

**Figure 8:**
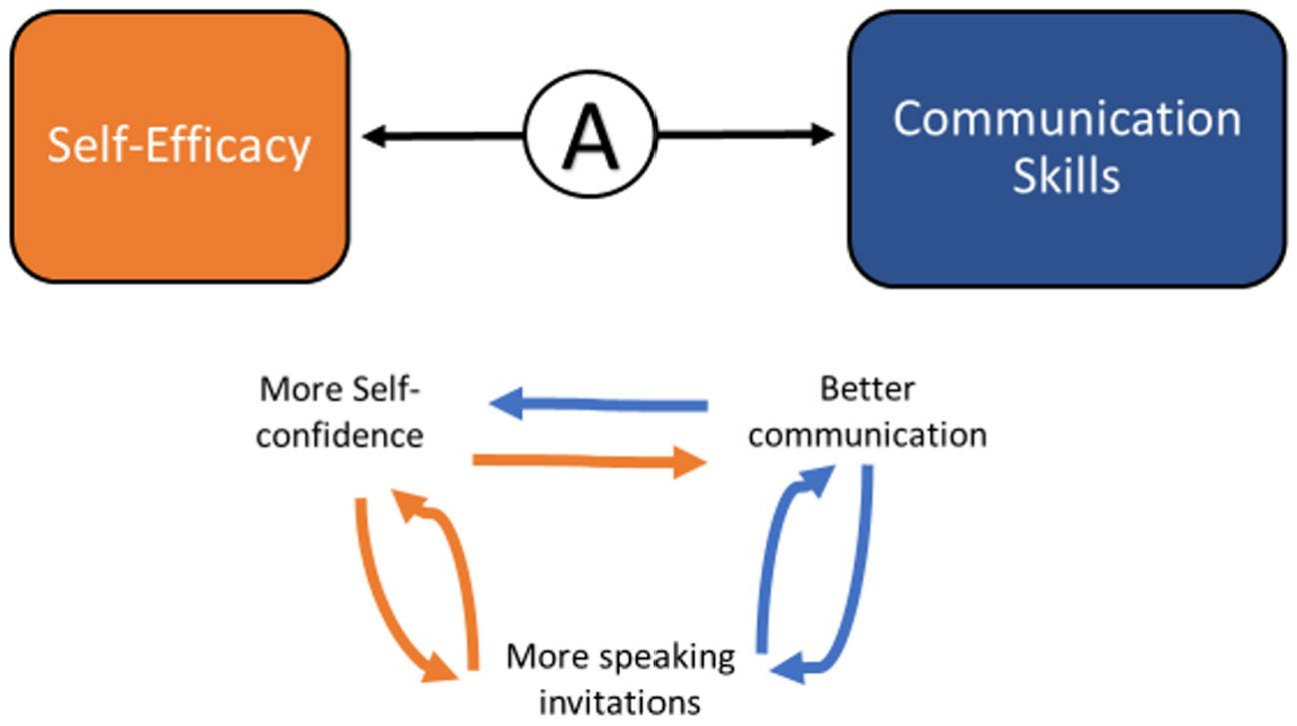
The interaction between self-efficacy and communication skills.

An important association that emerged was the increased self-confidence that FLEX members felt with improved communication, such as public speaking abilities. In 18 interviews with FLEX graduates who mentioned increased confidence, half of them also mentioned their improved speaking abilities. The reverse was also observed, where success at public speaking gave interviewees increased self-confidence. This interdependent relationship is an exemplar of reciprocal determinism posited by social cognitive theory (Wood and Bandura, 1989), resulting in a feedback loop and increased sophistication of communication skills (Figure 8).

*“I can much more comfortably speak in front of a crowd of a variety of sizes, and I probably would not be able to do that as easily if I had not done the [training]… that process was very, very challenging for me. The whole watching, giving a short speech, telling an impromptu story, watching yourself on video… I was terribly uncomfortable during the entirety of it, but it was really valuable and really important and it’s helped me with …a presentation for our national annual conference for my professional body…. I don’t think I would have gotten to this point if it hadn’t been for FLEX.” (FE28) (See also FE102 under Self-efficacy: Self-awareness and Self-confidence)*.

#### FLEX Graduates Engaged Their Colleagues More Productively and Learned to “Flex” Styles

**Figure 9:**
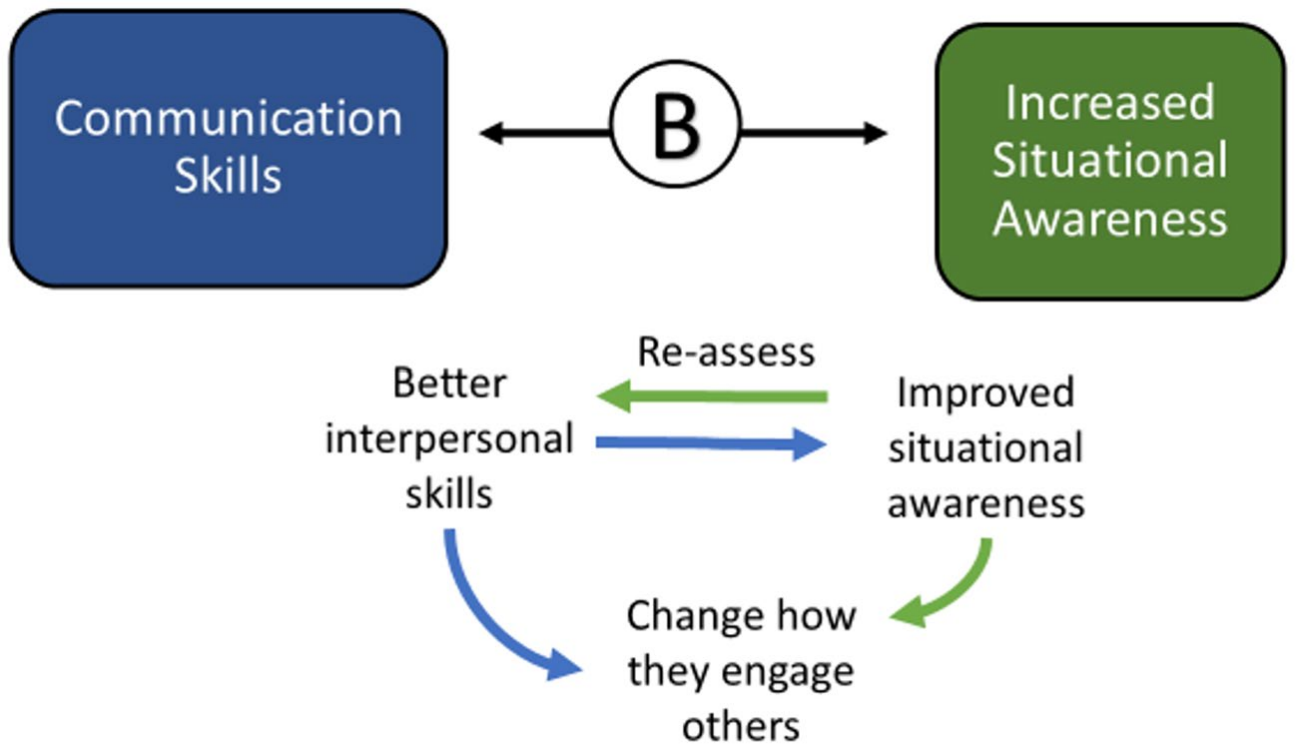
The interaction between communication skills and increased situational awareness.

Improvements in interpersonal skills development helped graduates both inwardly evaluate their situational awareness and outwardly demonstrate a change in the way they communicate in particular situations. This also resulted in greater self-confidence and their colleagues took notice, helping them to achieve leadership positions (Figure 9).

*“I think that going through the conflict resolution training and thinking through the value process of who I am and what I do was very helpful to process that time period that we went through in our department…But I’ve had a serious conversation afterward trying to come to a closer working relationship again, and I think that FLEX gave me skills and tools to do it in a good way.” (FE102) (See also FE28 and FE11, above, Increased Situational Awareness: Organizational awareness, being aware of your own management style and learning to “flex” styles)*.

#### FLEX Graduates Advocated for Themselves and for Others and Developed a Pay-it-Forward Mentality

**Figure 10:**
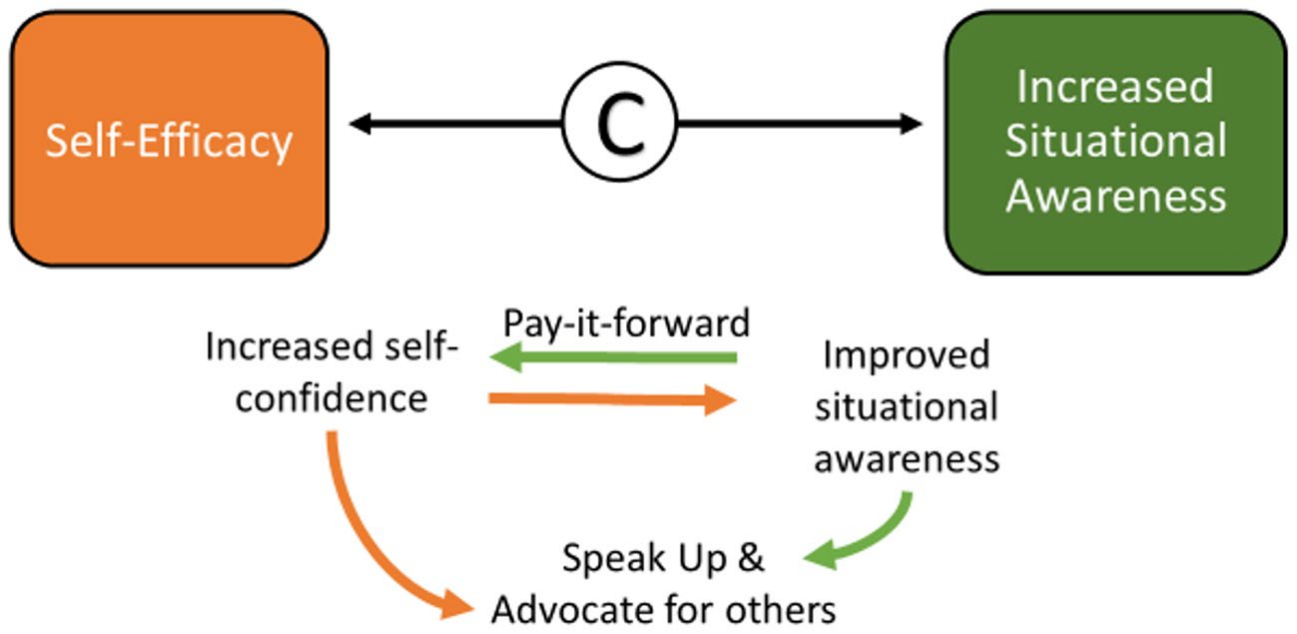
The interaction between self-efficacy and increased situational awareness.

As FLEX participants developed greater self-confidence, they began to think about and engage differently with a greater situational awareness that the environment could be made better for everyone. This led to FLEX members advocating for peers and for improving healthcare, whether they received credit or not, in a “pay-it-forward” mentality *(See FE62, above, under Increased situational awareness: Pay-it-forward; See FE28, above, under Increased Situational Awareness: Organizational awareness, being aware of your own management style and learning to “flex” styles; Figure 10)*.

#### FLEX Members Show How They Value Networking by Mentoring, Teaching the Next Generation of Trainees

**Figure 11:**
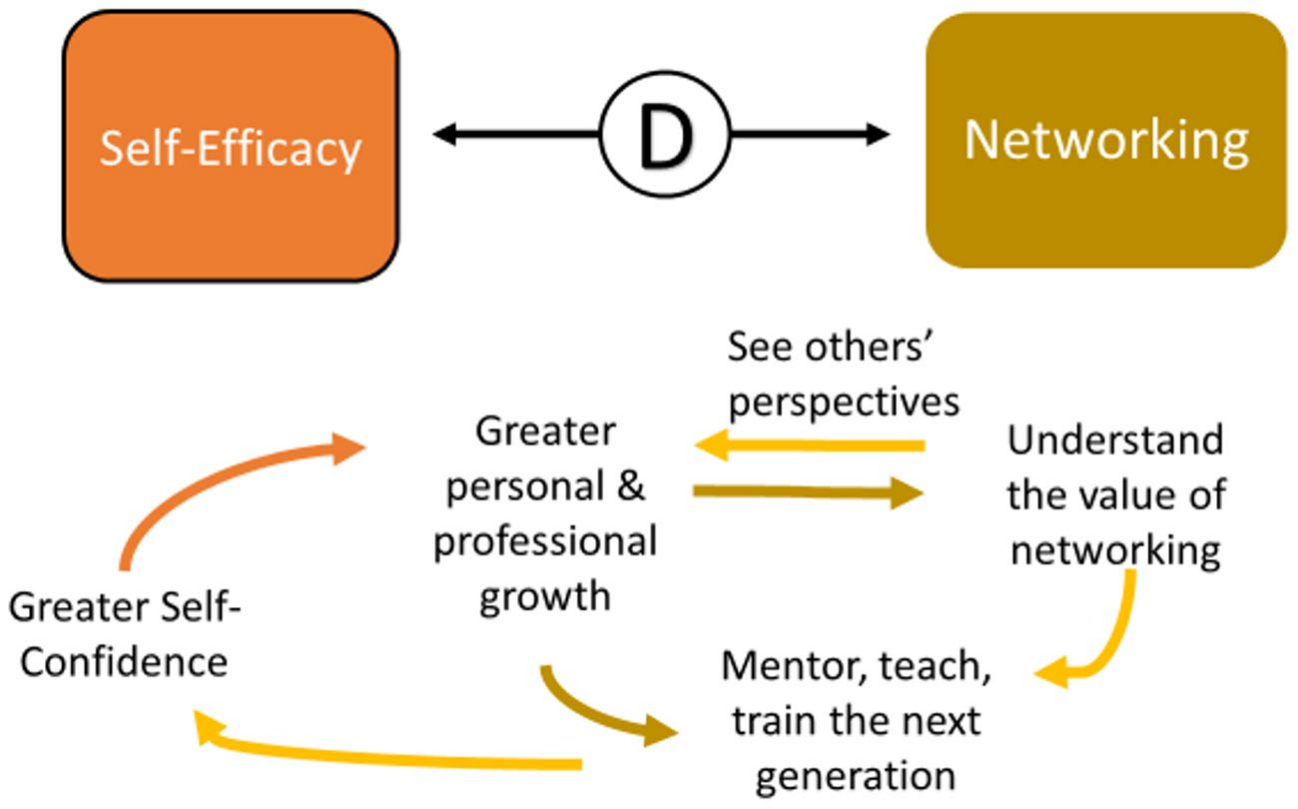
The interaction between self-efficacy and networking.

Once FLEX participants began to appreciate the high value in networking, they took what they had learned back to their own environments and expanded their mentoring, teaching, and training of the next generation of trainees. Similarly, networking exposed them to different perspectives, which allowed them to show both personal and professional growth. Training the next generation also gave them greater self-confidence, which also promoted their personal growth (See FE71, above, under Networking: Understanding the value of networking; See FE66, above, under Networking: Understanding the value of networking; Figure 11).

#### FLEX Graduates Envisioned New Career Directions and Took on Greater Leadership Rolls

**Figure 12:**
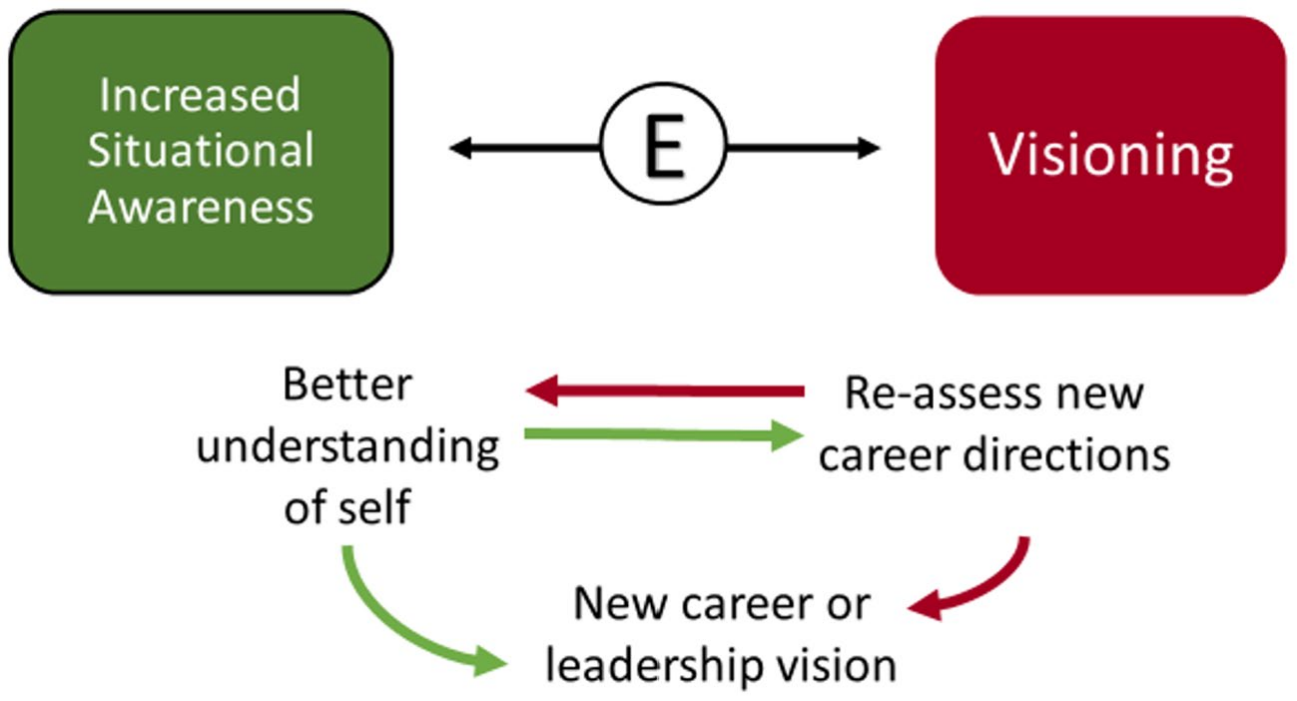
The interaction between increased situational awareness and visioning.

As FLEX participants developed increased professional situational awareness, that new understanding led many of the women to engage in visioning and re-assess their career and examine potentially new career directions. In many cases, those new directions involved greater responsibility and expanded leadership activities *(See FE71-2 quotes-, above, under Visioning, or forward-looking personal introspection: Re-evaluating priorities; See FE15 and FE11, above, under Visioning, or forward-looking personal introspection: Developing a career vision; Figure 12)*.

#### FLEX Participants Developed Improved Relational Capacity With an Expanded Network That Helped Them to Re-Evaluate Their Priorities and Goals

**Figure 13:**
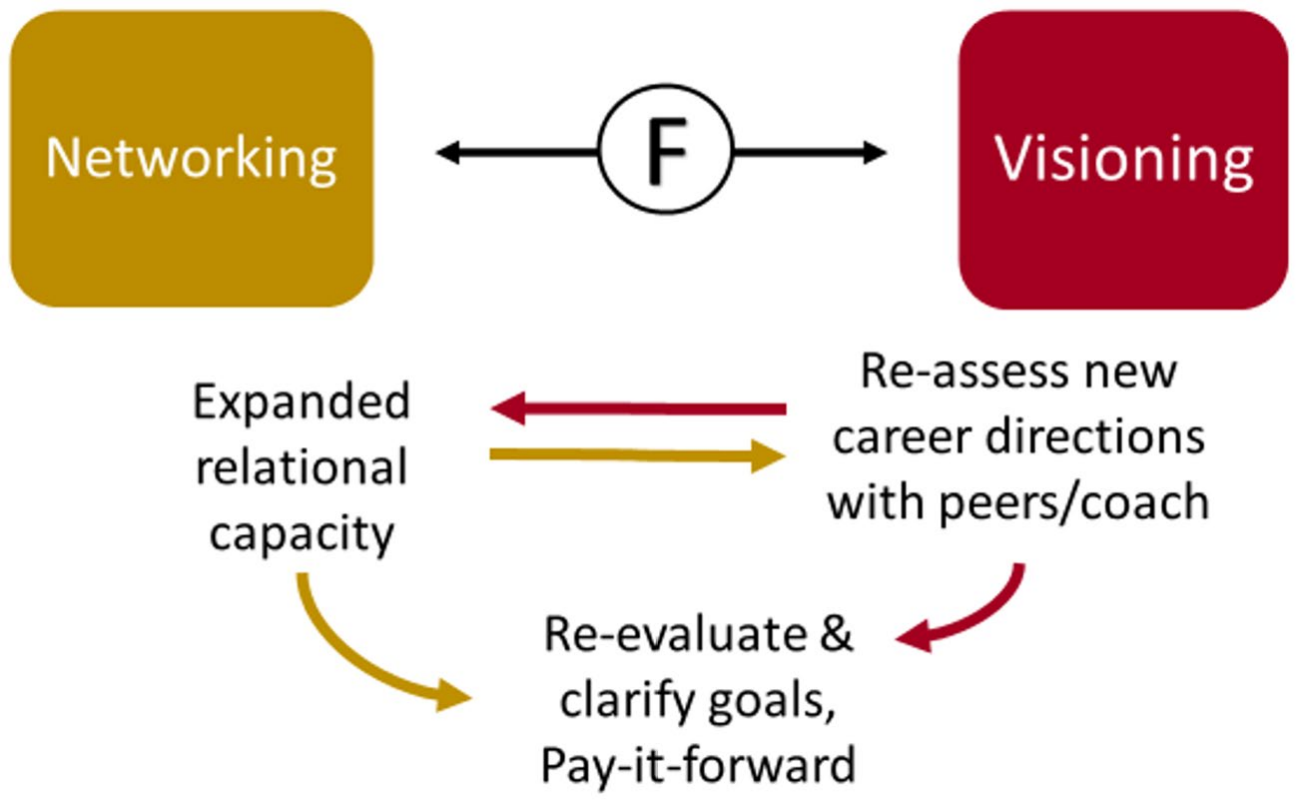
The interaction between networking and visioning.

Clarifying their career vision as women in medicine, with the help of peers and the coach, helped many FLEX graduates to see the importance of teaching the value of networking to the next generation of trainees *(See FE95, above, under Networking: Understanding the value of networking; See FE66, above, under Networking: Understanding the value of networking; See FE84, above, under Self-Efficacy: Enhanced professional visibility; Figure 13)*.

## Strengths, Limitations, and Future Directions

Strengths of this study include the extremely diverse representation of the FLEX respondents. Interviewees included women faculty who had worked between 2 and 26 years at their institutions and came from every academic rank, institutional affiliation, type of academic degree, and cultural background. Qualitative data were from multiple data sources, including interviews, application forms, pre- and post-program surveys and provided multiple ways to corroborate evidence. The resulting qualitative analysis provides other programs with several valuable constructs to examine leadership training outcomes that may be of interest to them. This study also highlights the significant value that small-cohort, long-term leadership training provides in terms of the strong relationships and trust that forms among participants and the persistently high relational capacity that confers among members—with each other and with their colleagues, trainees, and mentees.

For limitations, the interviews were not from a random sample of FLEX participants and thus, they are not necessarily representative of all FLEX participants’ experiences. Likewise, not all interviews were equally informative. Purposive sampling was used to select respondents who agreed to be interviewed and who represented a highly diverse sampling of FLEX graduates to ensure the broadest possible types of responses. Interview questions were designed based on the program logic model to obtain respondents’ opinions on specific features and outcomes of the program. Although this may have limited the scope of responses somewhat, it was mitigated by having several other data sources and collection methods such as both fixed- and open-response survey questions, program data and application data from participants.

Leadership development programs for women are considered only one aspect of improving the numbers of women in leadership positions. Conventionally, academic medicine has approached gender equity from the bottom up, with much of the expectation for improving conditions for women being placed upon the women themselves. Institutions need to take responsibility for making structural changes to improve the working environment in academic medicine and several solutions have been proposed. To promote a culture conducive to academic success for women, institutions should prioritize the following dimensions: equal access to resources and opportunities, work-life balance, freedom from gender biases, and supportive leadership (Westring et al., 2012); raise awareness about gender inequalities and promote diversity in all levels of committees and panels (Butkus et al., 2018); provide interventions that interrupt both conscious and unconscious biases (Carnes et al., 2015); counsel women on wage differences and training early-career females to negotiate salary and to ask for more transparency (ASH Clinical News, 2018); and mentor and sponsor women (Ayyala et al., 2019). Finally, training grants and research granting institutions must pay close attention to the recruitment and training of women as well as offer interventions to assist women with family care responsibilities (Jagsi et al., 2007).

In conclusion, this study suggests that the enduring benefits of the FLEX Program include improved communication skills, expanded situational awareness, and relational capacity, greater self-efficacy and self-confidence, improved networking and an understanding of the value of networking. FLEX is rooted in principles of intentional change theory, leadership assessment and development and coaching with compassion contained in contemporary management literature streams. FLEX was a transformative experience when participants articulated their leadership vision and developed a plan to execute their goals toward attaining leadership roles. All these factors led FLEX graduates to have greater visibility and more speaking invitations and to engage with their colleagues more effectively. Similarly, FLEX graduates could better advocate for themselves and for others as well as paying it forward to mentor and train the next generation of faculty. Finally, participants learned the value of re-evaluating their goals and their career vision to be able to envision themselves in greater leadership rolls. The factors that strongly influenced behavior change: communication skills, situational awareness, self-efficacy, networking and visioning, provide valuable constructs for other programs to evaluate following leadership development training. Each emergent factor contains sub-themes that are characterized in social learning theory, social network theory, and resonant leadership. These results suggest factors influencing behavior change are generalizable for leadership training. Future studies include examining leadership position attainment, personal goal attainment, accomplishments, and using the measure of Leader Self and Means Efficacy (Hannah et al., 2012) to articulate changes in leadership self-efficacy.

## Data Availability Statement

The raw data supporting the conclusions of this article will be made available by the authors, without undue reservation.

## Ethics Statement

This study involved human participants, and it was reviewed and approved by the Case Western Reserve University Institutional Review Board (IRB-2018-2243). Written informed consent for participation was not required for this study in accordance with the national legislation (45 CFR § 46.116, paragraph f) and institutional requirements. Participants provided verbal consent to participate in this study.

## Author Contributions

CP and PC envisioned the study’s overall design. SK and BE coordinated participant recruitment and facilitated access to archival FLEX Program data. CP conducted the interviews, co-coded certain transcripts, and co-performed data analysis to affirm the reliability of data analysis. JG transcribed the audio interviews, coded the transcripts, and performed data analysis. CP and JG drafted the manuscript. All authors contributed to the article and approved the submitted version.

## Funding

This study received two grants (2016 and 2018) totaling $6,750 Academic Careers in Engineering & Science (ACES) Advanced Opportunity Grant from Case Western Reserve University to help defray some costs of data analysis and participant remuneration. The ACES program is made available with the generous support of the National Science Foundation (NSF; Grant #0245054). This project (CP) was supported by the Clinical and Translational Science Collaborative of Cleveland which is funded by the National Institutes of Health (NIH), National Center for Advancing Translational Science (NCATS), and Clinical and Translational Science Award (CTSA) grant, UL1TR002548. The content is solely the responsibility of the authors and does not necessarily represent the official views of the NIH.

## Conflict of Interest

The authors declare that the research was conducted in the absence of any commercial or financial relationships that could be construed as a potential conflict of interest.

## Publisher’s Note

All claims expressed in this article are solely those of the authors and do not necessarily represent those of their affiliated organizations, or those of the publisher, the editors and the reviewers. Any product that may be evaluated in this article, or claim that may be made by its manufacturer, is not guaranteed or endorsed by the publisher.

## Footnotes

^1^PREPO stands for Point, Reason, Evidence, Point, Outcome and is an acronym to remember an easy and effective way to give a short impromptu speech. Copyright © 2015 VOICE-PRO INC®.
