# Supplementary figures and images for: Breaking Through Barriers: Factors That Influence Behavior Change Toward Leadership for Women in Academic Medicine
Source: Front Psychol. 2022 May 13;13:854488. doi: 10.3389/fpsyg.2022.854488 (PMC9136302; doi:10.3389/fpsyg.2022.854488)

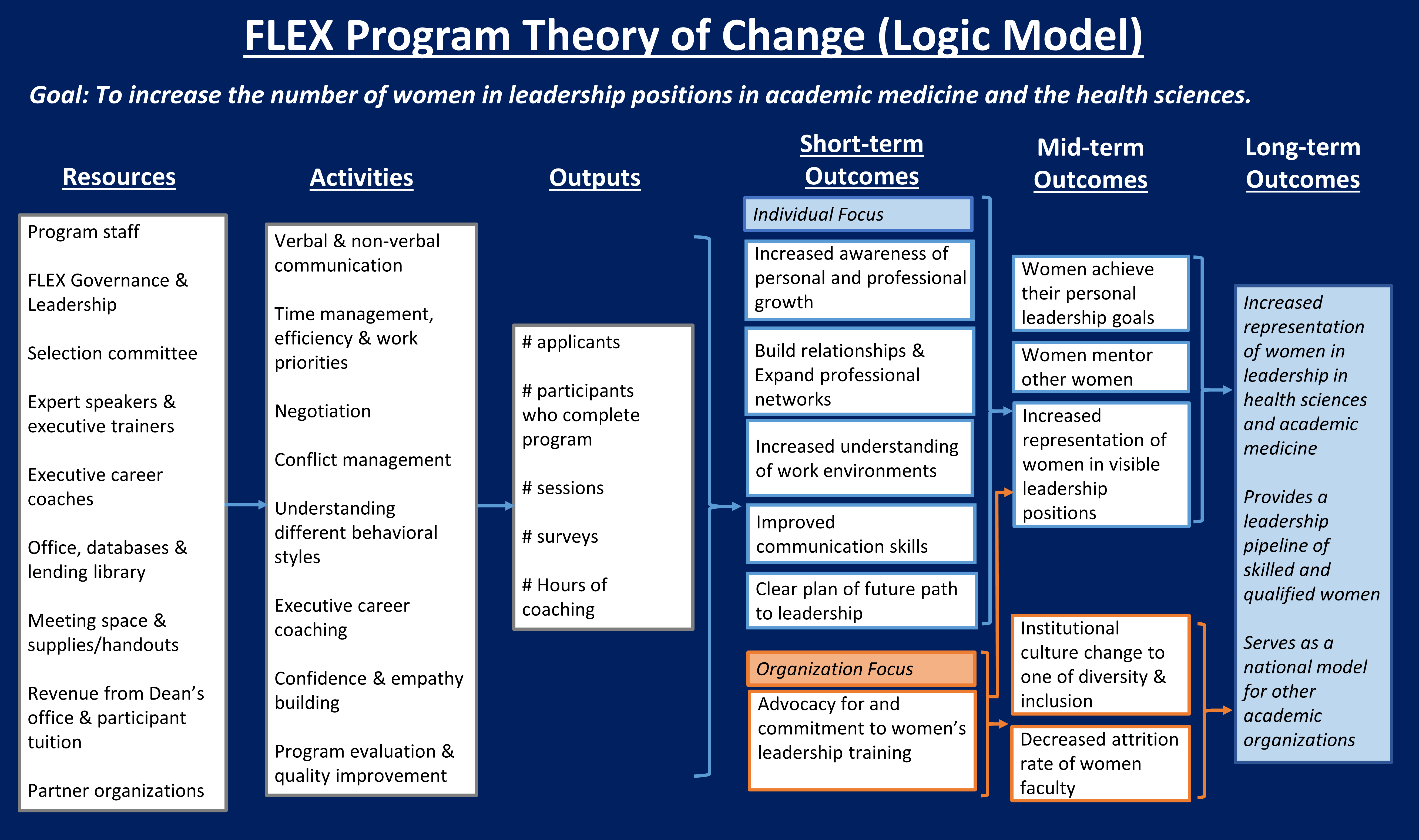

Supplement: Supplementary file 1 [file Image_14.png]
